# Supplementary material for: Giant five-photon absorption from multidimensional core-shell halide perovskite colloidal nanocrystals
Source: Nat Commun. 2017 May 12;8:15198. doi: 10.1038/ncomms15198 (PMC5437305; doi:10.1038/ncomms15198)
Supplement: Supplementary Information — Supplementary Figures, Supplementary Notes and Supplementary References [file ncomms15198-s1.pdf]

## Supplementary Figures

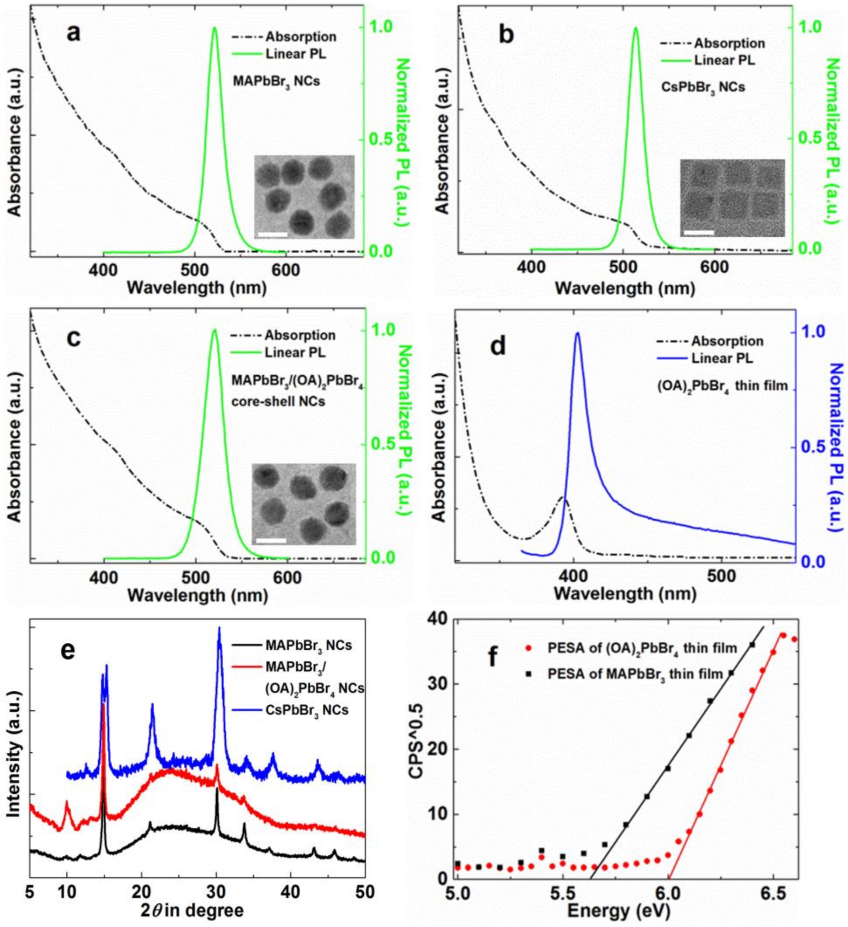


**Supplementary Figure 1 | 1PA spectra and one-photon excited PL spectra, TEM images, XRD patterns of the perovskite NCs and PESA measurements.** (**a-c**) 1PA spectra and one-photon excited PL spectra of the $MAPbBr_3$, $CsPbBr_3$ and $MAPbBr_3/(OA)_2PbBr_4$ NCs, respectively, together with their representative TEM images (scale bar, 10 nm) shown in the inset; (**d**) 1PA spectra and one-photon excited PL spectra of the $(OA)_2PbBr_4$ thin film; (**e**) XRD patterns of the $MAPbBr_3$, $MAPbBr_3/(OA)_2PbBr_4$ and $CsPbBr_3$ NCs; (**f**) PESA results from the $MAPbBr_3$ and $(OA)_2PbBr_4$ thin films.

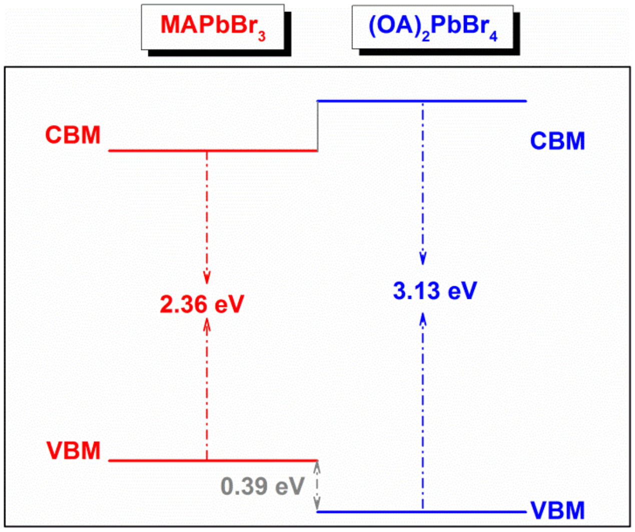


**Supplementary Figure 2 | The band offset of $MAPbBr_3$/$(OA)_2PbBr_4$.** The VBM difference (0.39 eV) is from theoretical calculations and the band gaps are experimental values.

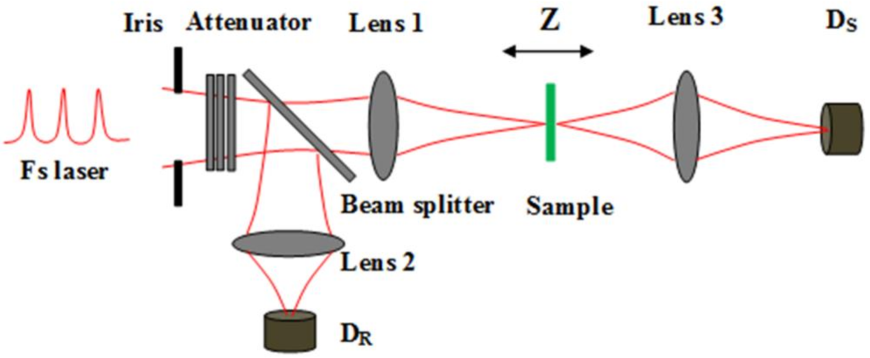


**Supplementary Figure 3 | Schematic of the open-aperture *Z*-scan set-up.** $D_S$ and $D_R$ are the signal power detector (RkP 465, Laser Probe) and reference power detector (RkP 465, Laser Probe), respectively.

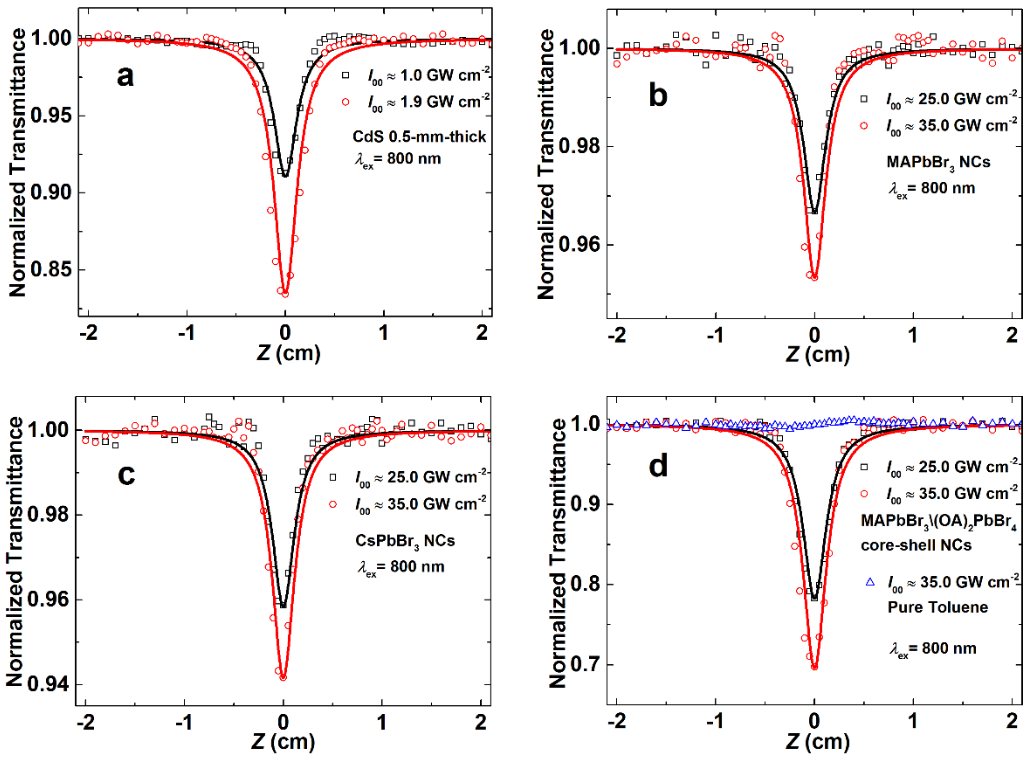


**Supplementary Figure 4 | Open-aperture *Z*-scan measurements.** (**a**) Open-aperture *Z*-scan curves of the standard sample CdS (0.5-mm thick) at 800 nm with peak excitation intensities of ~ 1.0 and ~ 1.9 GW cm$^{-2}$; (**b-d**) Open-aperture *Z*-scan responses from the toluene solutions of $MAPbBr_3$ (~ 2.0 μM), $MAPbBr_3/(OA)_2PbBr_4$ (~ 2.1 μM) and $CsPbBr_3$ NCs (~ 1.0 μM) contained in 1-mm-thick cuvette under laser excitation at 800 nm and with peak intensities of ~ 25.0 and ~ 35.0 GW cm$^{-2}$. The almost flat open-aperture *Z*-scan curve of the toluene under the same excitation condition (contained in 1-mm-thick cuvette, laser excitation at 800 nm with peak intensity of ~ 35.0 GW cm$^{-2}$) is also shown in (**d**).

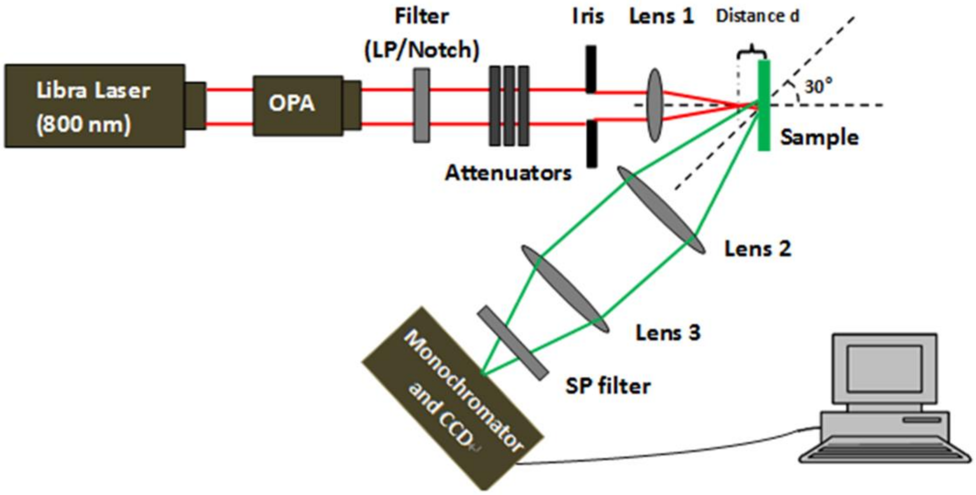


**Supplementary Figure 5 | Experimental setup for MEPL measurements.** The excitation wavelengths were varied from 675 nm to 2300 nm to investigate two-, three-, four- and five-photon absorption excited PL. OPA, LP and SP are abbreviations for optical parametric amplifier, long-pass filter and short-pass filter. For excitation wavelengths smaller than 800 nm, a notch filter at 800 nm was utilized to block off the light at undesired wavelengths. For excitation wavelengths larger than 800 nm, an appropriate long-pass filter (at 750 nm or at 1500 nm) was applied to filter out light at unwanted wavelengths. A short-pass filter at 650 nm was applied to filter out the excitation laser beam from the signal.

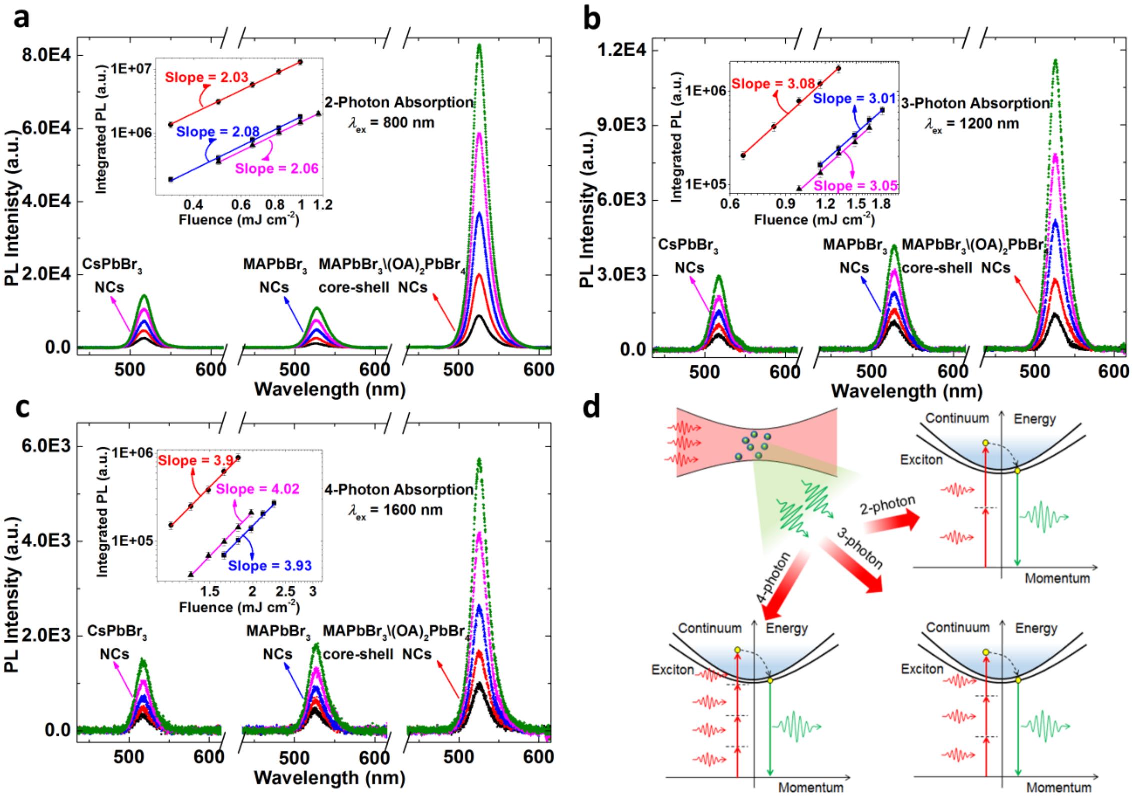


**Supplementary Figure 6 | Two, three, and four-photon excited upconversion PL spectra from toluene solution of perovskite NCs.** Insets indicate their excitation fluence dependence by the log-log plots of spectrally integrated PL intensity vs excitation laser fluences. (**a-c**) Two-, three- and four-photon-absorption excited PL spectra from toluene solutions of $MAPbBr_3$ NCs (~ 2.0 μM), $MAPbBr_3/(OA)_2PbBr_4$ NCs (~ 2.1 μM) and $CsPbBr_3$ NCs (~ 1.0 μM) excited by fs laser beam at 800, 1200, 1600 nm, respectively. Insets are their corresponding quadratic, cubic and quartic dependence on the excitation fluence; (**d**) Schematic illustrating the processes of 2PPL, 3PPL and 4PPL in the perovskite NCs corresponding to (**a - c**).

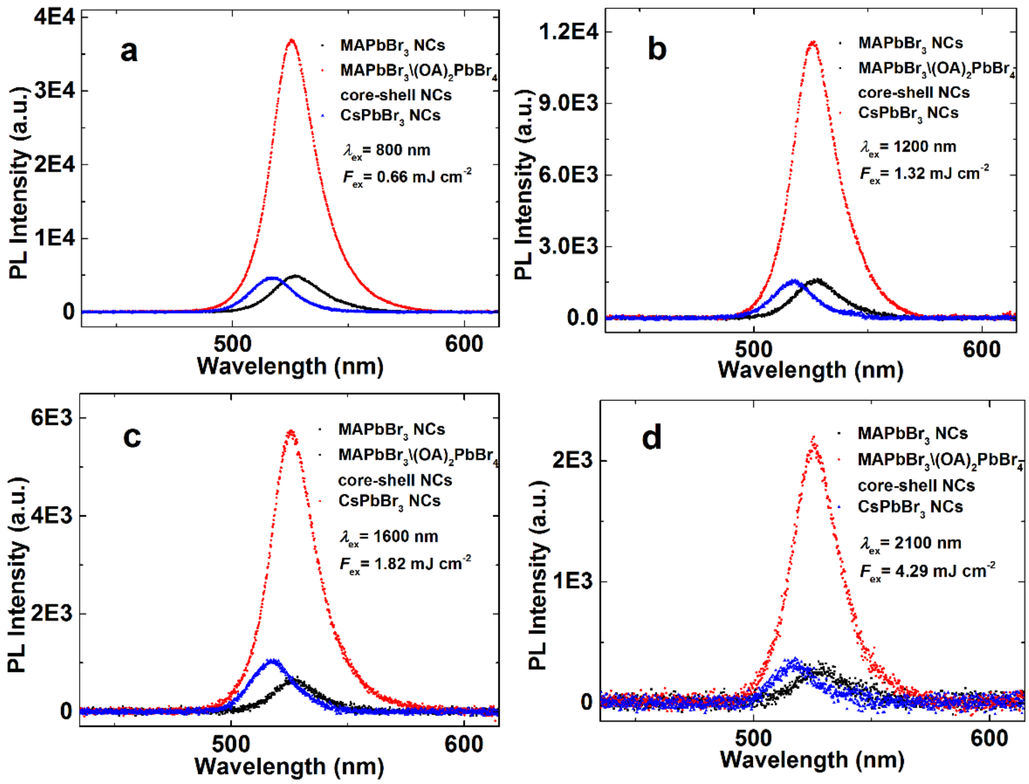


**Supplementary Figure 7 | Comparison of the MEPL (2, 3, 4, and 5-photon) from the toluene solution of perovskite NCs excited at 800, 1200, 1600, and 2100 nm.** (**a**) Comparison of the two-photon-excited frequency-upconverted PL at 800 nm from the core-only $MAPbBr_3$ NCs (~ 2.0 μM), core-shell $MAPbBr_3/(OA)_2PbBr_4$ NCs (~ 2.1 μM) and $CsPbBr_3$ NCs (~ 1.0 μM) under the same excitation fluence of ~ 0.66 mJ $cm^{-2}$; (**b**) Comparison of the three-photon-excited frequency-upconverted PL at 1200 nm from the perovskite NCs under the excitation fluence of ~ 1.32 mJ $cm^{-2}$; (**c**) Comparison of the four-photon-excited frequency-upconverted PL at 1600 nm from the perovskite NCs under the excitation fluence of ~ 1.82 mJ $cm^{-2}$; (**d**) Comparison of the five-photon-excited frequency-upconverted PL at 2100 nm from the perovskite NCs under the excitation fluence of ~ 4.29 mJ $cm^{-2}$.

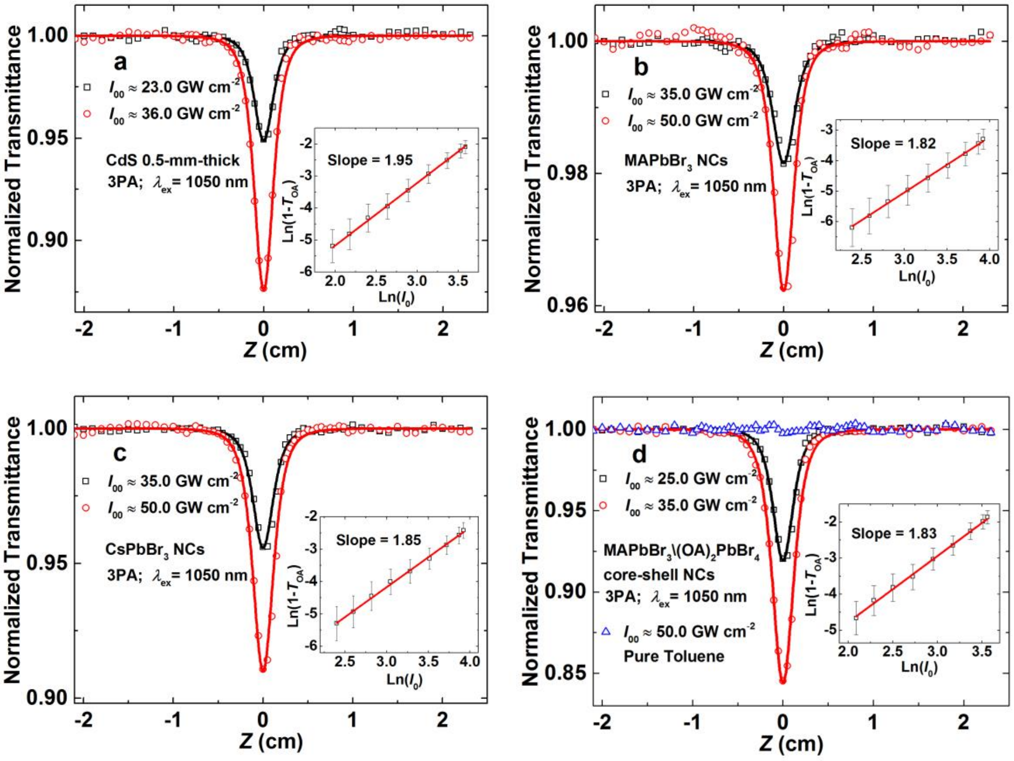


**Supplementary Figure 8 | Open-aperture *Z*-scan measurements on 3PA in perovskite NCs at 1050 nm.** (**a**) Open-aperture *Z*-scan curves of the standard sample CdS (0.5-mm thick) at 1050 nm with excitation peak intensities of ~23.0 and ~36.0 GW cm$^{-2}$; (**b-c**) Open-aperture *Z*-scan responses from the toluene solutions of $MAPbBr_3$ (~2.0 μM) and $CsPbBr_3$ NCs (~1.0 μM) contained in 1-mm-thick cuvette under laser excitation at 1050 nm and with peak intensities of ~35.0 and ~50.0 GW cm$^{-2}$; (**d**) Open-aperture *Z*-scan responses from the toluene solutions of $MAPbBr_3/(OA)_2PbBr_4$ (~2.1 μM) contained in 1-mm-thick cuvette excited at 1050 nm with peak intensities of ~25.0 and ~35.0 GW cm$^{-2}$. The almost flat open-aperture Z-scan curve of the toluene under the same excitation condition (contained in 1-mm-thick cuvette, laser excitation at 1050 nm with peak intensity of ~50.0 GW cm$^{-2}$) is also shown in (**d**).

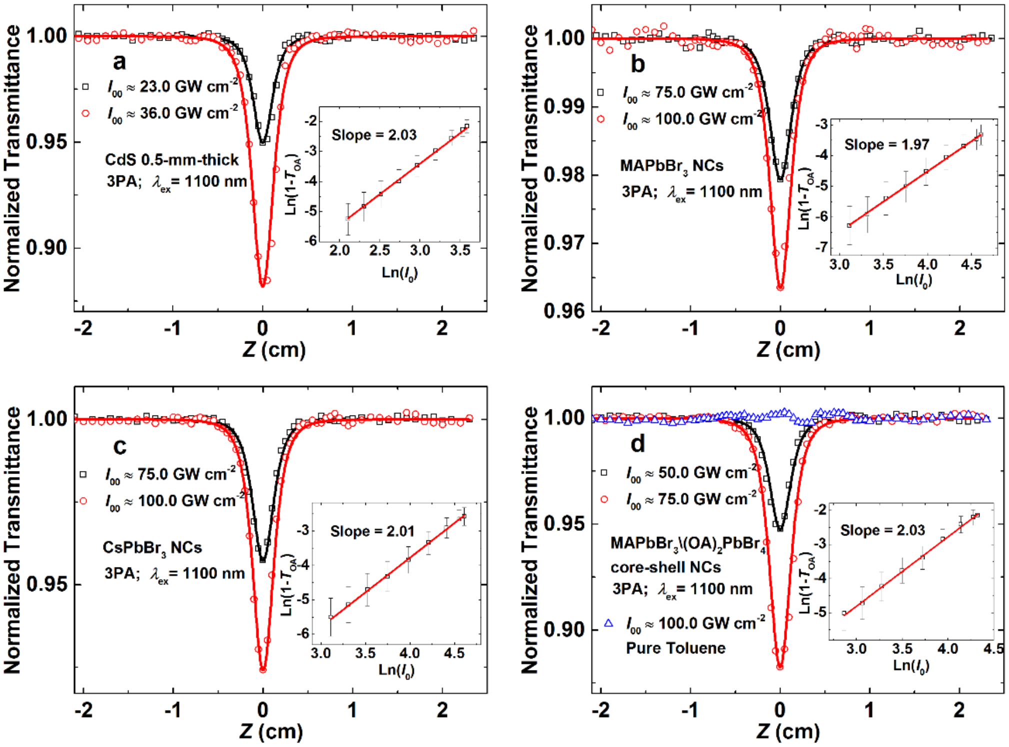


**Supplementary Figure 9 | Open-aperture *Z*-scan measurements on 3PA in perovskite NCs at 1100 nm.** (**a**) Open-aperture *Z*-scan curves of the standard sample CdS (0.5-mm thick) at 1100 nm with excitation peak intensities of ~23.0 and ~36.0 GW cm$^{-2}$; (**b-c**) Open-aperture Z-scan responses from the toluene solutions of $MAPbBr_3$ (~2.0 μM) and $CsPbBr_3$ NCs (~1.0 μM) contained in 1-mm-thick cuvette under laser excitation at 1100 nm and with peak intensities of ~75.0 and ~100.0 GW cm$^{-2}$; (**d**) Open-aperture *Z*-scan responses from the toluene solutions of $MAPbBr_3/(OA)_2PbBr_4$ (~2.1 μM) contained in 1-mm-thick cuvette excited at 1100 nm with peak intensities of ~50.0 and ~75.0 GW cm$^{-2}$. The almost flat open-aperture *Z*-scan curve of the toluene under the same excitation condition (contained in 1-mm-thick cuvette, laser excitation at 1100 nm with peak intensity of ~100.0 GW cm$^{-2}$) is also shown in (**d**).

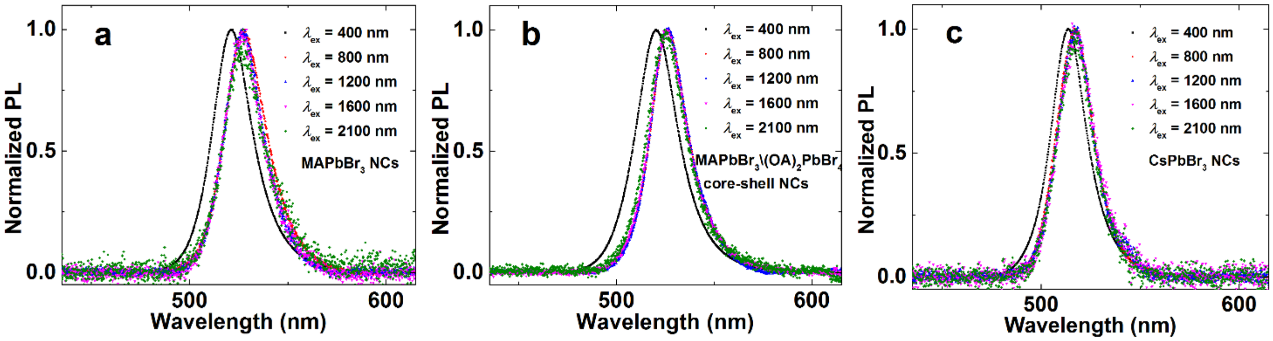


**Supplementary Figure 10 | Comparison of the normalized one-photon-excited and multi-photon-excited PL spectra from perovskite NCs.** (**a-c**) Comparision of the normalized one-photon-excited and multi-photon-excited PL spectra from $MAPbBr_3$, $MAPbBr_3/(OA)_2PbBr_4$ and $CsPbBr_3$ NCs, respectively.

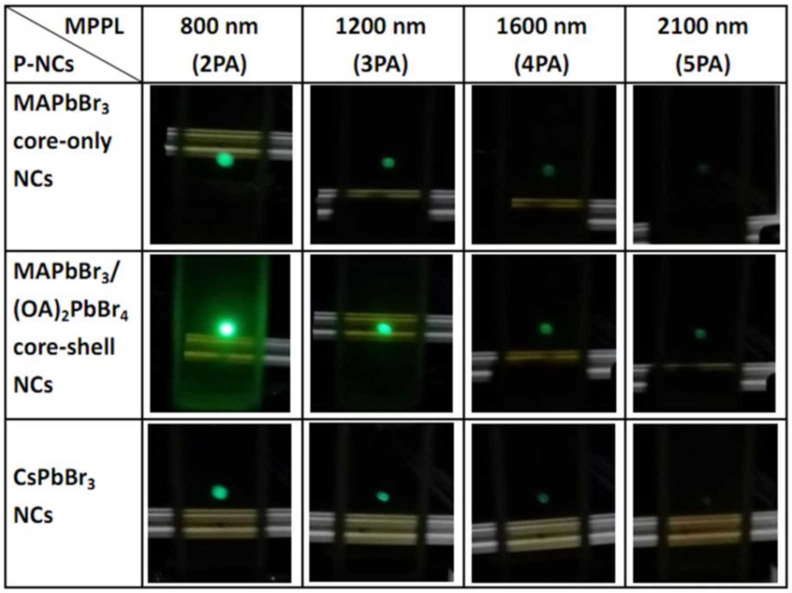


**Supplementary Figure 11 | Photographs of luminescence from $MAPbBr_3$, $MAPbBr_3/(OA)_2PbBr_4$ and $CsPbBr_3$ NCs at 800, 1200, 1600 and 2100 nm femtosecond laser excitation.**

| Samples \ Excitation fluence | 3.63 mJ $cm^{-2}$ Excited at 2100 nm | 4.62 mJ $cm^{-2}$ Excited at 2100 nm | 5.66 mJ $cm^{-2}$ Excited at 2100 nm |
|---|---|---|---|
| R6G in methanol (~ 2.1 μM) | 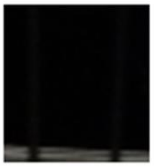 | 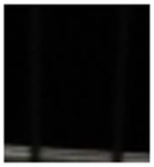 | 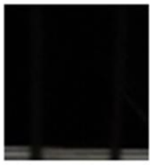 |
| $MAPbBr_3/(OA)_2PbBr_4$ NCs in toluene (~2.1 μM) | 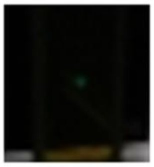 | 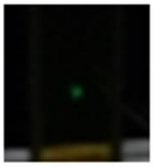 | 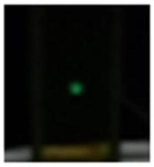 |

**Supplementary Figure 12 | Comparison of the photographs of five-photon excited upconversion luminescence from $MAPbBr_3/(OA)_2PbBr_4$ NCs in toluene with that from R6G in methanol at 2100 nm femtosecond laser excitation and under the same experimental conditions.**

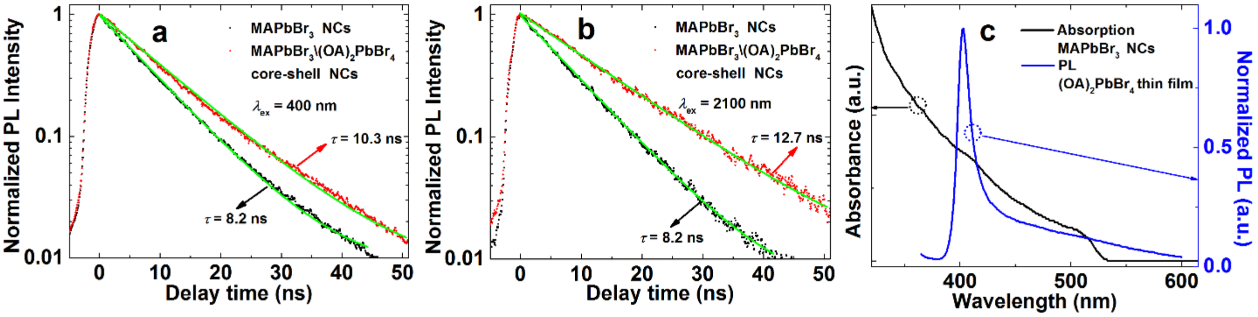


**Supplementary Figure 13 | Comparison between the PL decay traces of the $MAPbBr_3$ and $MAPbBr_3/(OA)_2PbBr_4$ NCs and the spectral overlap between shell emission and core absorption.** (**a**) Comparison between PL decay curves from $MAPbBr_3$ and $MAPbBr_3/(OA)_2PbBr_4$ NCs under one-photon excitation; (**b**) Longer five-photon excited PL lifetime of core-shell $MAPbBr_3/(OA)_2PbBr_4$ NCs as compared to the core-only $MAPbBr_3$ NCs. (**c**) Large spectral overlap between the emission of $(OA)_2PbBr_4$ thin film and the excitation of $MAPbBr_3$ NCs.

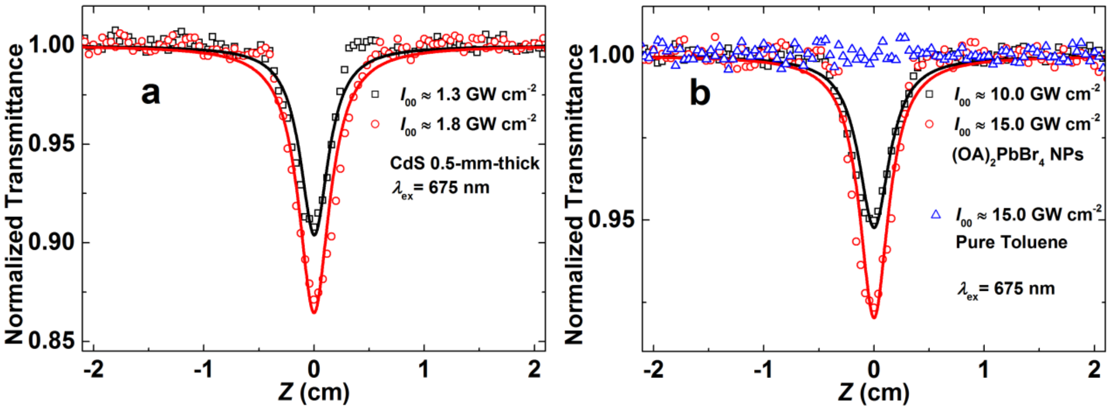


**Supplementary Figure 14 | Open-aperture *Z*-scan measurements on $(OA)_2PbBr_4$ NPs.** (**a**) Open-aperture *Z*-scan curves of the standard sample CdS (0.5-mm thick) at 675 nm with peak excitation intensities of ~1.3 and ~1.8 GW cm$^{-2}$; (**b**) Open-aperture *Z*-scan responses from the toluene solutions of $(OA)_2PbBr_4$ NPs (unknown concentration), contained in 1-mm-thick cuvette under laser excitation at 675 nm and with peak intensities of ~10.0 and ~15.0 GW cm$^{-2}$. The almost flat open-aperture *Z*-scan curve of the toluene under the same excitation condition (contained in 1-mm-thick cuvette, laser excitation at 675 nm with peak intensity of ~15.0 GW cm$^{-2}$) is also shown in (**b**).

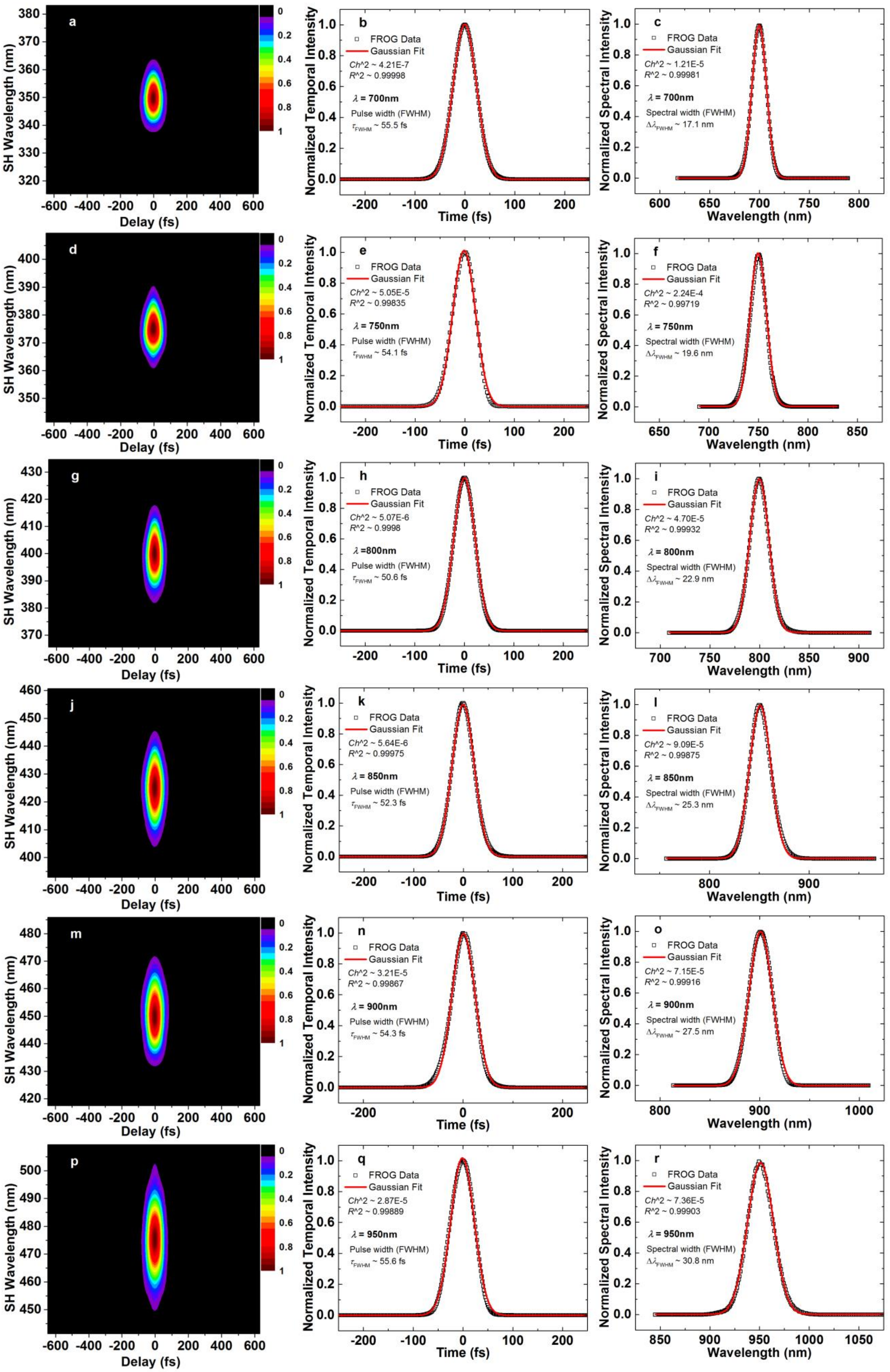
a
SH Wavelength (nm)
Delay (fs)
b
Normalized Temporal Intensity
FROG Data
Gaussian Fit
Ch^2 ~ 4.21E-7
R^2 ~ 0.99998
λ = 700nm
Pulse width (FWHM)
τFWHM ~ 55.5 fs
Time (fs)
c
Normalized Spectral Intensity
FROG Data
Gaussian Fit
Ch^2 ~ 1.21E-5
R^2 ~ 0.99981
λ = 700nm
Spectral width (FWHM)
ΔλFWHM ~ 17.1 nm
Wavelength (nm)
d
SH Wavelength (nm)
Delay (fs)
e
Normalized Temporal Intensity
FROG Data
Gaussian Fit
Ch^2 ~ 5.05E-5
R^2 ~ 0.99835
λ = 750nm
Pulse width (FWHM)
τFWHM ~ 54.1 fs
Time (fs)
f
Normalized Spectral Intensity
FROG Data
Gaussian Fit
Ch^2 ~ 2.24E-4
R^2 ~ 0.99719
λ = 750nm
Spectral width (FWHM)
ΔλFWHM ~ 19.6 nm
Wavelength (nm)
g
SH Wavelength (nm)
Delay (fs)
h
Normalized Temporal Intensity
FROG Data
Gaussian Fit
Ch^2 ~ 5.07E-6
R^2 ~ 0.9998
λ =800nm
Pulse width (FWHM)
τFWHM ~ 50.6 fs
Time (fs)
i
Normalized Spectral Intensity
FROG Data
Gaussian Fit
Ch^2 ~ 4.70E-5
R^2 ~ 0.99932
λ = 800nm
Spectral width (FWHM)
ΔλFWHM ~ 22.9 nm
Wavelength (nm)
j
SH Wavelength (nm)
Delay (fs)
k
Normalized Temporal Intensity
FROG Data
Gaussian Fit
Ch^2 ~ 5.64E-6
R^2 ~ 0.99975
λ = 850nm
Pulse width (FWHM)
τFWHM ~ 52.3 fs
Time (fs)
l
Normalized Spectral Intensity
FROG Data
Gaussian Fit
Ch^2 ~ 9.09E-5
R^2 ~ 0.99875
λ = 850nm
Spectral width (FWHM)
ΔλFWHM ~ 25.3 nm
Wavelength (nm)
m
SH Wavelength (nm)
Delay (fs)
n
Normalized Temporal Intensity
FROG Data
Gaussian Fit
Ch^2 ~ 3.21E-5
R^2 ~ 0.99867
λ = 900nm
Pulse width (FWHM)
τFWHM ~ 54.3 fs
Time (fs)
o
Normalized Spectral Intensity
FROG Data
Gaussian Fit
Ch^2 ~ 7.15E-5
R^2 ~ 0.99916
λ = 900nm
Spectral width (FWHM)
ΔλFWHM ~ 27.5 nm
Wavelength (nm)
p
SH Wavelength (nm)
Delay (fs)
q
Normalized Temporal Intensity
FROG Data
Gaussian Fit
Ch^2 ~ 2.87E-5
R^2 ~ 0.99889
λ = 950nm
Pulse width (FWHM)
τFWHM ~ 55.6 fs
Time (fs)
r
Normalized Spectral Intensity
FROG Data
Gaussian Fit
Ch^2 ~ 7.36E-5
R^2 ~ 0.99903
λ = 950nm
Spectral width (FWHM)
ΔλFWHM ~ 30.8 nm
Wavelength (nm)

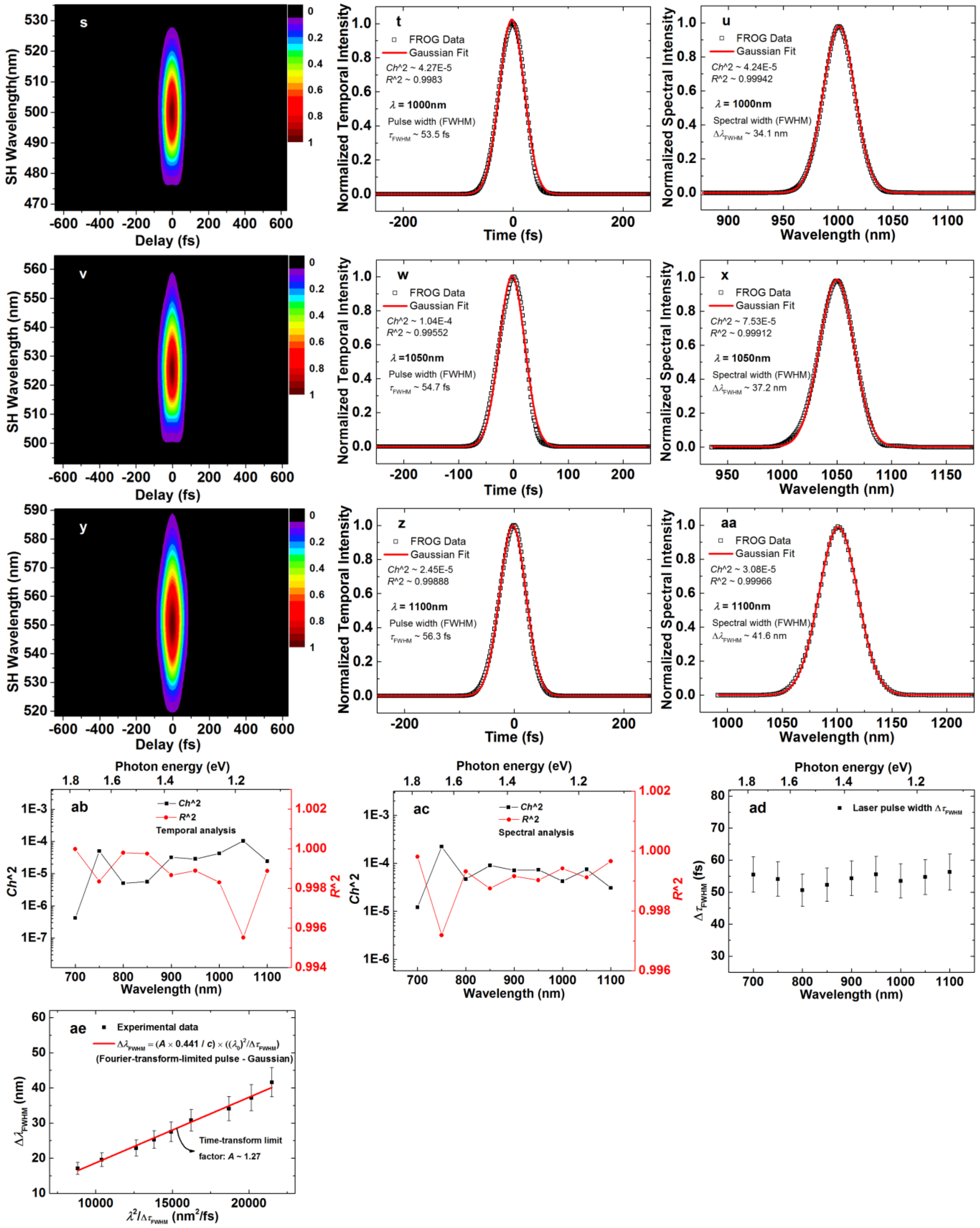


**Supplementary Figure 15 | Pulse-width and spectral bandwidth measurements of the laser beam at wavelength range 700-1100 nm by FROG.** (**a, d, g, j, m, p, s, v, y**) Measured two-dimensional FROG traces of the laser pulses. *x* axis represents the delay time and *y* axis corresponds to the wavelength. The right side is the scale bar characterizing the normalized magnitude of the FROG signal; (**b, e, h, k, n, q, t, w, z**) The corresponding temporal distributions; (**c, f, i, l, o, r, x, aa**) The corresponding spectral distributions; (**ab**) Summarized *Ch*^2 and *R*^2 of the Gaussian function fitting to laser pulse temporal distribution at all wavelengths. Both the negligible *Ch*^2 and the very close to unity *R*^2 demonstrates the good fitting thus indicating the Gaussian temporal distribution of the laser pulses; (**ac**) Summarized *Ch*^2 and *R*^2 of the Gaussian function fitting to spectral distribution at all wavelengths with similar negligible *Ch*^2 and very close to unity *R*^2,

demonstrating the good fitting thus the Gaussian spectral distribution of the laser pulses, which is in agreement with (**ab**); (**ad**) Summary of the measured pulse-widths of the laser pulses at all wavelengths; (**ae**) Relation between the measured spectral bandwidths and temporal pulse-widths, together with the fitting with the time-bandwidth relation of Fourier-transform-limited pulses: $\Delta\lambda_{\mathrm{FWHM}} = (A \times K / c) \times ((\lambda_0)^2 / \Delta\tau_{\mathrm{FWHM}})$.

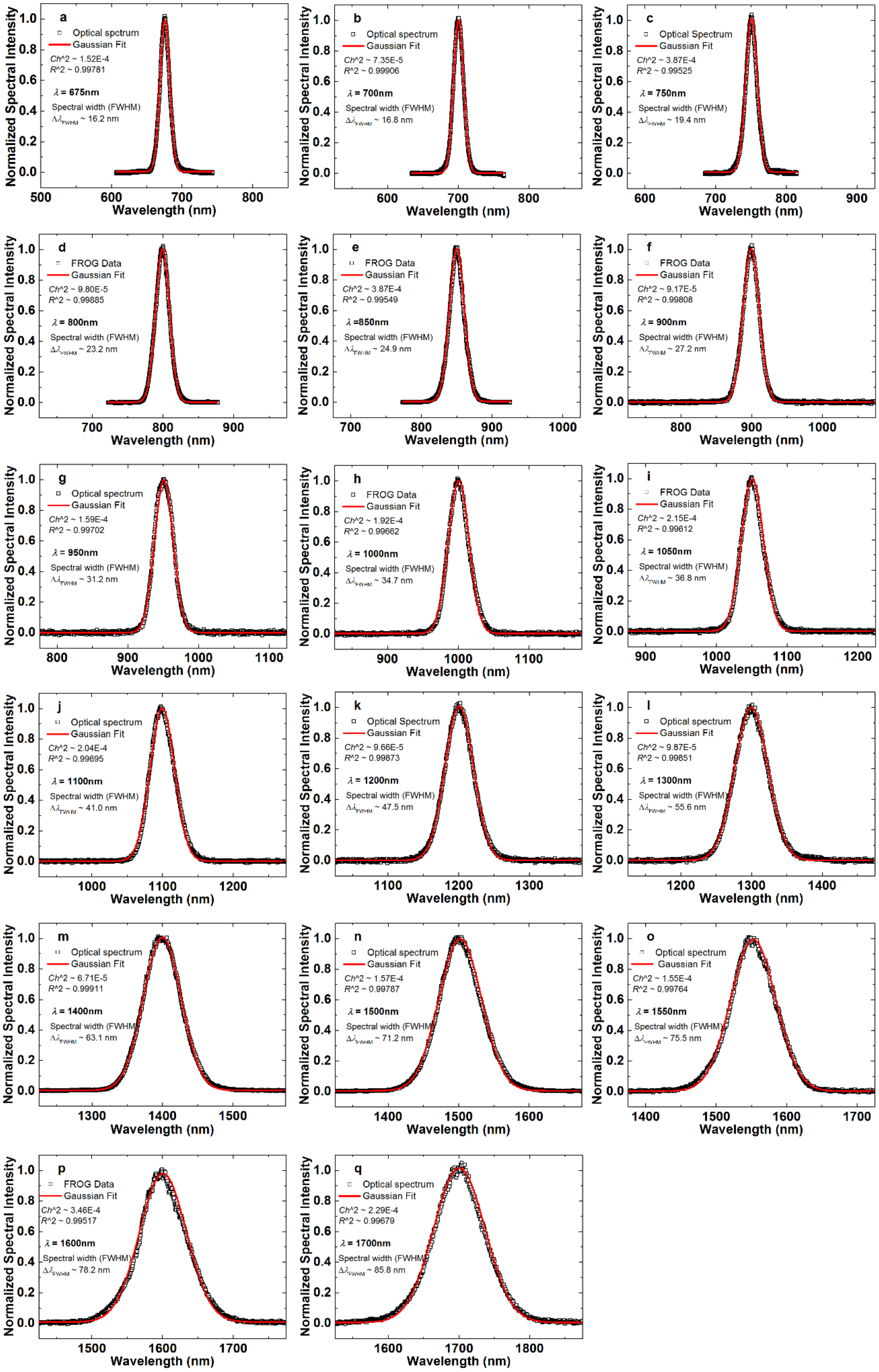
a
Optical spctrum
Gaussian Fit
Ch^2 ~ 1.52E-4
R^2 ~ 0.99781
λ = 675nm
Spectral width (FWHM)
Δλ_FWHM ~ 16.2 nm
b
Optical spectrum
Gaussian Fit
Ch^2 ~ 7.35E-5
R^2 ~ 0.99906
λ = 700nm
Spectral width (FWHM)
Δλ_FWHM ~ 16.8 nm
c
Optical Spectrum
Gaussian Fit
Ch^2 ~ 3.87E-4
R^2 ~ 0.99525
λ = 750nm
Spectral width (FWHM)
Δλ_FWHM ~ 19.4 nm
d
FROG Data
Gaussian Fit
Ch^2 ~ 9.80E-5
R^2 ~ 0.99885
λ = 800nm
Spectral width (FWHM)
Δλ_FWHM ~ 23.2 nm
e
FROG Data
Gaussian Fit
Ch^2 ~ 3.87E-4
R^2 ~ 0.99549
λ =850nm
Spectral width (FWHM)
Δλ_FWHM ~ 24.9 nm
f
FROG Data
Gaussian Fit
Ch^2 ~ 9.17E-5
R^2 ~ 0.99808
λ = 900nm
Spectral width (FWHM)
Δλ_FWHM ~ 27.2 nm
g
Optical spectrum
Gaussian Fit
Ch^2 ~ 1.59E-4
R^2 ~ 0.99702
λ = 950nm
Spectral width (FWHM)
Δλ_FWHM ~ 31.2 nm
h
FROG Data
Gaussian Fit
Ch^2 ~ 1.92E-4
R^2 ~ 0.99662
λ = 1000nm
Spectral width (FWHM)
Δλ_FWHM ~ 34.7 nm
i
FROG Data
Gaussian Fit
Ch^2 ~ 2.15E-4
R^2 ~ 0.99612
λ = 1050nm
Spectral width (FWHM)
Δλ_FWHM ~ 36.8 nm
j
Optical spectrum
Gaussian Fit
Ch^2 ~ 2.04E-4
R^2 ~ 0.99695
λ = 1100nm
Spectral width (FWHM)
Δλ_FWHM ~ 41.0 nm
k
Optical Spectrum
Gaussian Fit
Ch^2 ~ 9.66E-5
R^2 ~ 0.99873
λ = 1200nm
Spectral width (FWHM)
Δλ_FWHM ~ 47.5 nm
l
Optical spectrum
Gaussian Fit
Ch^2 ~ 9.87E-5
R^2 ~ 0.99851
λ = 1300nm
Spectral width (FWHM)
Δλ_FWHM ~ 55.6 nm
m
Optical spectrum
Gaussian Fit
Ch^2 ~ 6.71E-5
R^2 ~ 0.99911
λ = 1400nm
Spectral width (FWHM)
Δλ_FWHM ~ 63.1 nm
n
Optical spectrum
Gaussian Fit
Ch^2 ~ 1.57E-4
R^2 ~ 0.99787
λ = 1500nm
Spectral width (FWHM)
Δλ_FWHM ~ 71.2 nm
o
Optical spectrum
Gaussian Fit
Ch^2 ~ 1.55E-4
R^2 ~ 0.99764
λ = 1550nm
Spectral width (FWHM)
Δλ_FWHM ~ 75.5 nm
p
FROG Data
Gaussian Fit
Ch^2 ~ 3.46E-4
R^2 ~ 0.99517
λ = 1600nm
Spectral width (FWHM)
Δλ_FWHM ~ 78.2 nm
q
Optical spectrum
Gaussian Fit
Ch^2 ~ 2.29E-4
R^2 ~ 0.99679
λ = 1700nm
Spectral width (FWHM)
Δλ_FWHM ~ 85.8 nm
Normalized Spectral Intensity
Wavelength (nm)

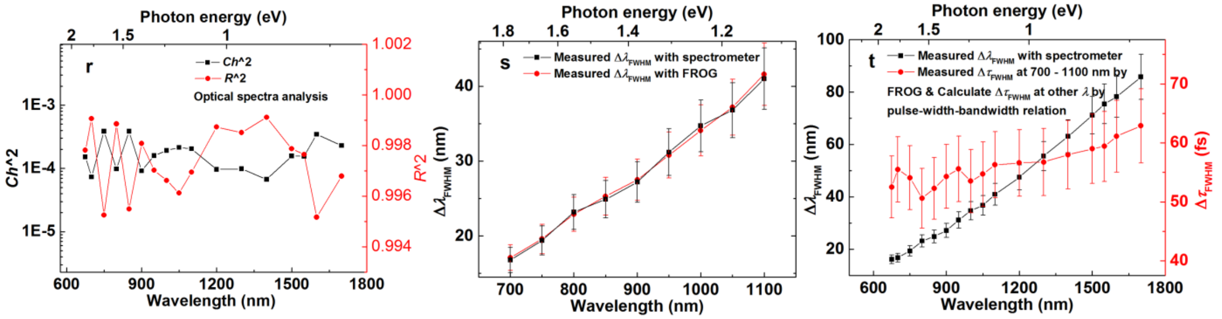


**Supplementary Figure 16 | Spectral bandwidth measurements of the laser beam at wavelength range 675-1700 nm by a visible monochromator coupled with silicon CCD and infrared monochromator coupled with liquid-nitrogen-cooled InGaAs infrared detector, together with the pulse derivation.** (**a-q**) Measured optical spectra at the wavelengths range from 675 nm to 1700nm; (**r**) Summarized *Ch*^2 and *R*^2 of the Gaussian function fitting to laser pulse spectral distribution at wavelengths 675-1700 nm. Both the negligible *Ch*^2 and the very close to unity *R*^2 demonstrate the good fitting, thus indicating the Gaussian spectral distribution of the laser pulses; (**s**) Comparison of the acquired spectral bandwidths at 700-1100 nm by spectrometer with that obtained from the FROG measurements, indicating high consistency with variation less than 5%; (**t**) Summary of the measured spectral bandwidths at 675-1700 nm and the comparison of the derived pulse-widths at wavelengths of 675 & 1200 - 1700 nm based on the pulse-width-bandwidth relation of Fourier-transform-limited pulses with the measured pulse-widths at 700-1100 nm by FROG, revealing the pulse widths at wavelength range 675 – 1700 nm are in the range of (50.6 ± 5.1) – (62.9 ± 6.3) fs, with variation smaller than 25%, consistent with the specification of the OperA-Solo OPA.

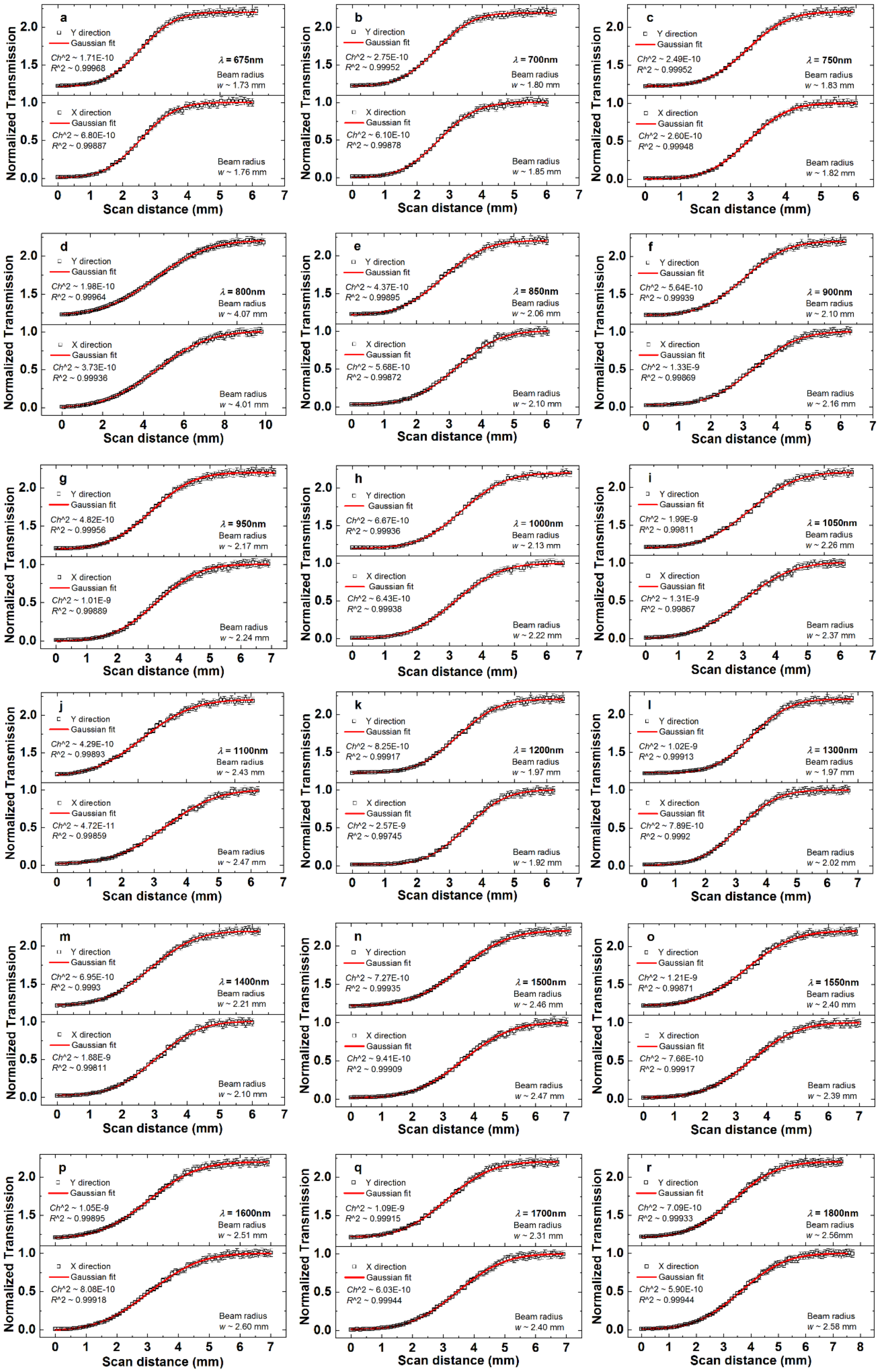
a
Y direction
Gaussian fit
Ch^2 ~ 1.71E-10
R^2 ~ 0.99968
λ = 675nm
Beam radius
w ~ 1.73 mm
X direction
Gaussian fit
Ch^2 ~ 6.80E-10
R^2 ~ 0.99887
Beam radius
w ~ 1.76 mm
b
Ch^2 ~ 2.75E-10
R^2 ~ 0.99952
λ = 700nm
w ~ 1.80 mm
Ch^2 ~ 6.10E-10
R^2 ~ 0.99878
w ~ 1.85 mm
c
Ch^2 ~ 2.49E-10
R^2 ~ 0.99952
λ = 750nm
w ~ 1.83 mm
Ch^2 ~ 2.60E-10
R^2 ~ 0.99948
w ~ 1.82 mm
d
Ch^2 ~ 1.98E-10
R^2 ~ 0.99964
λ = 800nm
w ~ 4.07 mm
Ch^2 ~ 3.73E-10
R^2 ~ 0.99936
w ~ 4.01 mm
e
Ch^2 ~ 4.37E-10
R^2 ~ 0.99895
λ = 850nm
w ~ 2.06 mm
Ch^2 ~ 5.68E-10
R^2 ~ 0.99872
w ~ 2.10 mm
f
Ch^2 ~ 5.64E-10
R^2 ~ 0.99939
λ = 900nm
w ~ 2.10 mm
Ch^2 ~ 1.33E-9
R^2 ~ 0.99869
w ~ 2.16 mm
g
Ch^2 ~ 4.82E-10
R^2 ~ 0.99956
λ = 950nm
w ~ 2.17 mm
Ch^2 ~ 1.01E-9
R^2 ~ 0.99889
w ~ 2.24 mm
h
Ch^2 ~ 6.67E-10
R^2 ~ 0.99936
λ = 1000nm
w ~ 2.13 mm
Ch^2 ~ 6.43E-10
R^2 ~ 0.99938
w ~ 2.22 mm
i
Ch^2 ~ 1.99E-9
R^2 ~ 0.99811
λ = 1050nm
w ~ 2.26 mm
Ch^2 ~ 1.31E-9
R^2 ~ 0.99867
w ~ 2.37 mm
j
Ch^2 ~ 4.29E-10
R^2 ~ 0.99893
λ = 1100nm
w ~ 2.43 mm
Ch^2 ~ 4.72E-11
R^2 ~ 0.99859
w ~ 2.47 mm
k
Ch^2 ~ 8.25E-10
R^2 ~ 0.99917
λ = 1200nm
w ~ 1.97 mm
Ch^2 ~ 2.57E-9
R^2 ~ 0.99745
w ~ 1.92 mm
l
Ch^2 ~ 1.02E-9
R^2 ~ 0.99913
λ = 1300nm
w ~ 1.97 mm
Ch^2 ~ 7.89E-10
R^2 ~ 0.9992
w ~ 2.02 mm
m
Ch^2 ~ 6.95E-10
R^2 ~ 0.9993
λ = 1400nm
w ~ 2.21 mm
Ch^2 ~ 1.88E-9
R^2 ~ 0.99811
w ~ 2.10 mm
n
Ch^2 ~ 7.27E-10
R^2 ~ 0.99935
λ = 1500nm
w ~ 2.46 mm
Ch^2 ~ 9.41E-10
R^2 ~ 0.99909
w ~ 2.47 mm
o
Ch^2 ~ 1.21E-9
R^2 ~ 0.99871
λ = 1550nm
w ~ 2.40 mm
Ch^2 ~ 7.66E-10
R^2 ~ 0.99917
w ~ 2.39 mm
p
Ch^2 ~ 1.05E-9
R^2 ~ 0.99895
λ = 1600nm
w ~ 2.51 mm
Ch^2 ~ 8.08E-10
R^2 ~ 0.99918
w ~ 2.60 mm
q
Ch^2 ~ 1.09E-9
R^2 ~ 0.99915
λ = 1700nm
w ~ 2.31 mm
Ch^2 ~ 6.03E-10
R^2 ~ 0.99944
w ~ 2.40 mm
r
Ch^2 ~ 7.09E-10
R^2 ~ 0.99933
λ = 1800nm
w ~ 2.56mm
Ch^2 ~ 5.90E-10
R^2 ~ 0.99944
w ~ 2.58 mm
Normalized Transmission
Scan distance (mm)

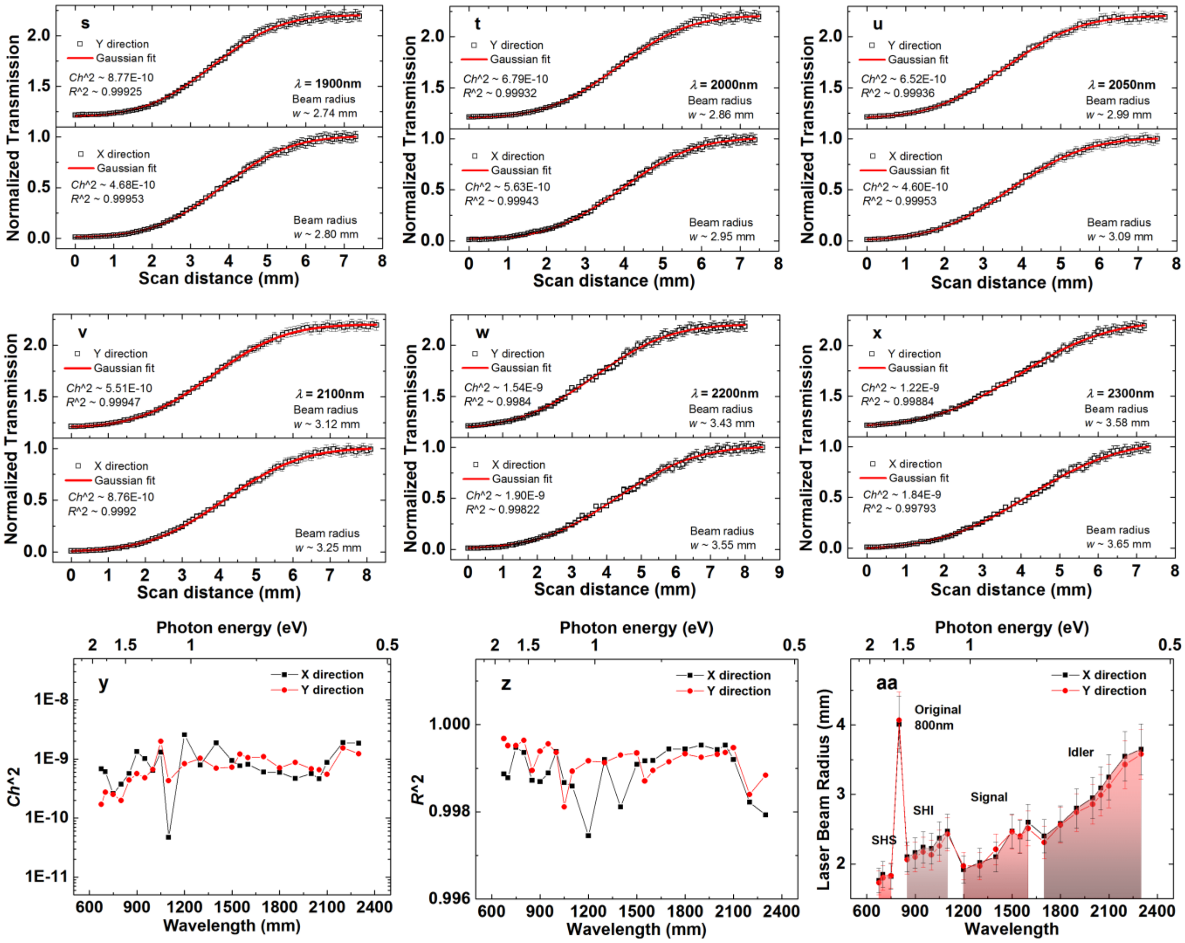


**Supplementary Figure 17 | Knife-edge scans along the *x*- and *y*-directions of the cross-sectional plane of the laser beam for characterizing the spatial distributions of the laser beam and measuring its beam radius.** (**a-x**) Measured knife-edge scans curves along the *x*- and *y*-directions at the excitation wavelengths range from 675 nm to 2300nm, together with the Gaussian distribution fitting; (**y,z**) Summarized *Ch*^2 and *R*^2 of the Gaussian fitting to laser spatial distributions along both *x*- and *y*-directions at wavelengths 675-2300 nm. Both the negligible *Ch*^2 and the very close to unity *R*^2 demonstrate the good fitting thus indicating the Gaussian spatial distribution of the laser beam at both *x*- and *y*-directions ; (**aa**) Summary of the measured laser beam radii along both *x*- and *y*-directions at 675-2300 nm. The high consistency of the measured laser beam radii in *x*-direction with that measured in *y*-direction at all wavelengths with variation less than 6 %, further validating that the two-dimension spatial profiles of the laser beams in the cross-sectional plane follow the Gaussian distributions.

# Supplementary Tables

**Supplementary Table 1.** Comparison between the measured 2PA cross-sections of $CsPbBr_3$ NCs (~ 9nm, cubic) at 800nm through *Z*-scan measurements to reported results excited at the same wavelength and measured with the same technique.

| | Xu, Y. *et al. J. Am. Chem. Soc.* **138**, 3761-3768 (2016) | Wang, Y. *et al. Nano Lett.* **16**, 448-453 (2016) | Lu, W.-G. *et al. Adv. Optical Mater.*, DOI:10.1002/adom.201600322 (2016) | Our results |
|---|---|---|---|---|
| $CsPbBr_3$ NCs | (~9 nm, cubic) | (~9 nm, cubic) | (~5 nm spherical and ~ 17 nm cubic) | (~9 nm, cubic) |
| Interpretation | Two-photon absorption (2PA) | Two-photon absorption (2PA) | Saturable absorption (SA) | Two-photon absorption (2PA) |
| 2PA cross-section at 800 nm ($\sigma_2$) | $2.7\times10^6$ GM | $1.2\times10^5$ GM | N.A. | $(2.0\pm0.3)\times10^6$GM |

**Supplementary Table 2.** Comparison of the *two-photon action cross-sections* of the $MAPbBr_3$, $MAPbBr_3/(OA)_2PbBr_4$, $CsPbBr_3$ NCs with the reported values from traditional semiconductor NCs and organic chromophores.

| Materials | Excitation laser source | PLQY | $\sigma_2$ ($10^6$GM) | $\eta\sigma_2$ ($10^6$GM) |
|---|---|---|---|---|
| $MAPbBr_3$ NCs in toluene (8-9 nm size, sphere) (our result) | 675 - 1000 nm; 50 fs; 1kHz | ~84% | $4.8 \times 10^{-1}$ – 6.2 (~$8.0\times 10^{-1}$ at 800 nm) | **~$4.1 \times 10^{-1}$ – 5.2 (~$6.7 \times 10^{-1}$ at 800 nm)** |
| $MAPbBr_3/(OA)_2PbBr_4$ NCs in toluene (9-10 nm size, sphere) (our result) | 675 - 1000 nm; 50 fs; 1kHz | ~92% | 3.3 – $4.0 \times 10^{1}$ (~5.0 at 800 nm) | **~3.0 – $3.7 \times 10^{1}$ (~4.6 at 800 nm)** |
| $CsPbBr_3$ NCs in toluene (~ 9 nm size, cube) (our result) | 675 - 1000 nm; 50 fs; 1kHz | ~55% | 1.8 – $2.4 \times 10^{1}$ (~2.0 at 800 nm) | **~$9.7 \times 10^{-1}$ – $1.3 \times 10^{1}$ (~1.1 at 800 nm)** |
| $CsPbBr_3$ NCs in toluene (~ 9 nm size, cube ) [2] | 800 nm; 100 fs; 1kHz | ~60% | 2.7 | **1.6** |
| $CsPbBr_3$ NCs in toluene (~ 9 nm size, cube ) [1] | 800 nm; 100 fs; 1kHz | N.A. | $1.2 \times 10^{-1}$ | **< $1.2 \times 10^{-1}$** |
| **II-VI group inorganic semiconductor NCs** | | | | |
| CdSe NCs in toluene (2.4 - 4.8 nm size) [53] | 800, 840 nm; 100fs; 82 MHz | 18% - 60% | $3.9 \times 10^{-4}$ – $1.0 \times 10^{-2}$ | **$7.5 \times 10^{-5}$ – $4.5 \times 10^{-3}$** |
| CdSe/ZnS NCs in water (11.2 nm size) [63] | 700 - 1000 nm; 100fs; 80MHz | 71% | $3.5 \times 10^{-2}$ – $6.6 \times 10^{-2}$ | **$2.5 \times 10^{-2}$ – $4.7 \times 10^{-2}$** |
| CdTe NCs in toluene (4.4 – 5.4 nm size) [53] | 840 nm; 100fs; 82MHz | 0.1% - 4.4% | $3.4 \times 10^{-3}$ – $8.0 \times 10^{-3}$ | **$8.0 \times 10^{-6}$ – $1.5 \times 10^{-4}$** |
| CdTe NCs in water (3 – 4 nm size) [64] | 720 - 950 nm; 100fs; 86 MHz | ~15% - 36% | ~$1.0 \times 10^{-3}$ – $1.8\times 10^{-2}$ | **~$1.5 \times 10^{-4}$ – $5.8 \times 10^{-3}$** |
| CdS NCs in chloroform (4.6 nm size) [54] | 770 - 890 nm; 100fs; 80 MHz | ~1.5% | ~$1.2 \times 10^{-4}$ – $1.8\times 10^{-3}$ | **~$1.8 \times 10^{-6}$– $2.7 \times 10^{-5}$** |
| ZnSe NCs in *n*-butanol (3.3 nm & 4.3 nm) [57] | 806 nm; 100 fs; 10Hz | ~ 2% | $4.9 \times 10^{-3}$ – $1.2 \times 10^{-2}$ | **$9.8\times 10^{-5}$ – $2.4 \times 10^{-4}$** |
| ZnSe/ZnS NCs in *n*-butanol (3.3 & 4.3 nm) [57] | 806 nm; 100 fs; 10Hz | 17% & 22% | $5.1 \times 10^{-3}$ – $1.4 \times 10^{-2}$ | **$1.1 \times 10^{-3}$ – $2.4 \times 10^{-3}$** |
| ZnS NCs in methanol (1.4 and 1.8 nm size) [26,65] | 532 & 520 nm; 28 ps; 10 Hz | ~ 0.5% | $1.7 \times 10^{-2}$ – $2.1 \times 10^{-2}$ | **$8.5 \times 10^{-5}$ – $1.1 \times 10^{-4}$** |
| **Large size CdSe/CdS dot in rod hetero-structure (2.4 nm size CdSe NC; 5 nm diameter, 39 and 180 nm length CdS nanorod) [44,60]** | 800 nm; 150 fs; 1 kHz | 56% & 8% | $2.3 \times 10^{-1}$ – 1.4 | **$1.1 \times 10^{-1}$ – $1.3 \times 10^{-1}$** |

| | | | | |
|---|---|---|---|---|
| **IV-VI group inorganic semiconductor NCs** | | | | |
| PbS NCs in toluene (3.0 – 5.3 nm size) [66,67] | 950 - 2400 nm; 140 fs; 1kHz; | ~ 20% – 55% | ~1.0 × $10^{-3}$ – 2.3 × $10^{-1}$ | **~ 5.5 × $10^{-4}$ – 4.6× $10^{-2}$** |
| PbS NCs in toluene (3.1 & 4.3nm size) [68] | 1120 - 2500 nm; 140 fs; 1kHz; | ~ 20% –55% | ~1.0 × $10^{-3}$ – 2.1× $10^{-2}$ | **~ 5.5 × $10^{-4}$ – 4.2× $10^{-3}$** |
| **III-V group inorganic semiconductor NCs** | | | | |
| InP/ZnS NCs in toluene (2.2 & 2.8 nm size) [69] | 800 nm; 100 fs; 1kHz | 58% &39% | 3.5 × $10^{-3}$ – 6.2× $10^{-3}$ | **2.0 × $10^{-3}$ – 2.4× $10^{-3}$** |
| InP/ZnS NCs in toluene (3.5 – 4.3 nm size) [70] | 750 - 1100 nm; 130fs; 1kHz | 31% – 40% | ~1.0 × $10^{-5}$ – 2.2× $10^{-3}$ | **4.0 × $10^{-6}$– 6.8× $10^{-4}$** |
| **VIII-IV group inorganic semiconductor NCs** | | | | |
| $Fe_3O_4$ in toluene (13 nm) [71] | 780 nm; 330fs; 1kHz | < 5% | 3.0 | **≲ 1.0 × $10^{-1}$** |
| **Organic molecules** | | | | |
| Bis(styryl)benzene derivatives with various structural motifs [33,72] | 518 - 945 nm; 100 fs; 1kHz | 0.9% – 90% | 2.1 × $10^{-4}$ –3.7× $10^{-3}$ | **3.1 × $10^{-5}$ – 3.2× $10^{-3}$** |
| Amine-terminated vyano-substituted phenylenevinylene oligomers [33,73] | 720 - 1000 nm; 90fs; 82MHz | 71% – 79% | ~8.0 × $10^{-4}$ – 5.3× $10^{-3}$ | **5.8 × $10^{-4}$ – 3.8× $10^{-3}$** |
| Symmetrical diphenylamino -fluorenes [33,74] | 590 -780 nm; 150fs; 1kHz | 40% – 100% | ~1.0 × $10^{-4}$ – 6.8× $10^{-3}$ | **~4 × $10^{-5}$ – 6.8× $10^{-3}$** |
| **Conjugated Polymers** | | | | |
| Poly[2-methoxy-5-(2-ethyl hexyloxy)-1,4-phenylenevi nylene] (MEH-PPV) [33,75] | 600 - 780 nm; 150fs; 1kHz | 8% | ~5.0 × $10^{-3}$ – 1.2× $10^{-2}$ | **~4.0 × $10^{-4}$ – 9.6× $10^{-4}$** |
| Methyl-substituted ladder-type poly(*p*-phenylene) (MeLPPP) [33,76,77] | 718 & 800 nm; 140fs; 1kHz | 30% | 1.8 × $10^{-2}$ & 4.0 × $10^{-2}$ | **5.4 × $10^{-3}$ &1.2 × $10^{-2}$** |
| dendrimers of 4,4'-bis (diphenylamino) stilbene (DPAS) [33,78] | 715 - 780 nm; ~150 fs; 76 MHz | 52% – 87% | 3.3 × $10^{-4}$ – 1.1 × $10^{-2}$ | **2.9 × $10^{-4}$ – 6.1 × $10^{-3}$** |
| **Fullerenes** | | | | |
| $C_{60}$(>DPAF-$C_9$) & | 775 nm; 160 fs; 1 kHz | N.A. | 3.1 × $10^{-5}$ & 8.2 × $10^{-5}$ | **<3.1 × $10^{-5}$ & 8.2 × $10^{-5}$** |

| | | | | |
|---|---|---|---|---|
| $C_{60}(>DPAF\text{-}C_9)_2$ [79] | | | | |
| Multiple [60]fullerene –Oligo(p-phenylene ethynylene) hybrids [80] | 800nm; 90fs; 1kHz | N.A. | $2.8 \times 10^{-5} – 1.4 \times 10^{-4}$ | **< $2.8 \times 10^{-5} – 1.4 \times 10^{-4}$** |
| **Metal complexes** | | | | |
| Nickel bis(dithiolene) complexes [81] | 1200 - 1580 nm; 140fs; 1 kHz | N.A. | ~$1.0 \times 10^{-5} – 5.3 \times 10^{-3}$ | **< $1.0 \times 10^{-5} – 5.3 \times 10^{-3}$** |
| Zn(II) or Cu(I) mono- and bis-(Schiff base) complexes [33,82] | 890nm; 100fs; 76 MHz | N.A. | $1.7 \times 10^{-3} – 1.1 \times 10^{-2}$ | **< $1.7 \times 10^{-3} – 1.1 \times 10^{-2}$** |
| Zn(II)-coordinated self-assembled extended 3D MLC [45] | 720 - 920 nm; 100fs; 80 MHz | 11% | ~$1.0 \times 10^{-4} – 2.4 \times 10^{-3}$ | **~$1.1 \times 10^{-5} – 2.6 \times 10^{-4}$** |
| **Porphyrins & Metallophophyrins** | | | | |
| Conjugated porphyrin dimers [33, 83] | 810 - 1000 nm; 100fs; 1 kHz | 5% – 10% | ~$1.0 \times 10^{-4} – 1.0 \times 10^{-2}$ | **~$1.0 \times 10^{-5} – 7.0 \times 10^{-4}$** |
| Zinc(II) Meso-Meso Linked Porphyrin Oligomers [33, 84] | 800 nm; 120fs; 5kHz | 4.5% – 11% | $4.5 \times 10^{-3} – 1.3 \times 10^{-2}$ | **$2.0 \times 10^{-4} – 1.4 \times 10^{-3}$** |
| **Biomolecules & Derivatives** | | | | |
| $\beta$-carotene [33,85] | 532 nm; 35 ps; 10 Hz | N.A. | $7.2 \times 10^{-4}$ | **< $7.2 \times 10^{-4}$** |
| $Fe^{2+}$ and $Fe^{3+}$ oxidation states of cytochrome c [33,86] | 560 - 850 nm; 120 fs; 1kHz | N.A. | ~$5.0 \times 10^{-5} – 1.1 \times 10^{-3}$ | **< $5.0 \times 10^{-5} – 1.1 \times 10^{-3}$** |

**Supplementary Table 3.** Comparison of the *three-photon action cross-sections* of the $MAPbBr_3$, $MAPbBr_3/(OA)_2PbBr_4$, $CsPbBr_3$ NCs with the reported values from traditional semiconductor NCs and organic chromophores.

| **Materials** | **Excitation laser source** | **PLQY** | **$\sigma_3$ ($10^{-74}cm^8s^3$ /photon$^3$)** | **$\eta\sigma_3$ ($10^{-74}cm^8s^3$ /photon$^3$)** |
|---|---|---|---|---|
| $MAPbBr_3$ NCs in toluene (8-9 nm size, sphere) (our result) | 1050 - 1500 nm; 50 fs; 1kHz | ~84% | $3.9 \times 10^{-1}$ – 3.3 | **~$3.3 \times 10^{-1}$ – 2.7** |
| $MAPbBr_3/(OA)_2PbBr_4$ NCs in toluene (9-10 nm size, sphere) (our result) | 1050 - 1500 nm; 50 fs; 1kHz | ~92% | 2.7 – $2.4 \times 10^{1}$ | **~2.5 – $2.2 \times 10^{1}$** |
| $CsPbBr_3$ NCs in toluene (~ 9 nm size, cube) (our result) | 1050 - 1500 nm; 50 fs; 1kHz | ~55% | $7.0 \times 10^{-1}$ – $1.4 \times 10^{1}$ | **~$3.8 \times 10^{-1}$ – 7.6** |
| **Inorganic semiconductor NCs** | | | | |
| CdSe NCs in hexane (2 - 4 nm size) [52,53] | 1300 nm; 160fs; 1 kHz | 20% - 50% | $7.2 \times 10^{-5}$ – $6.3 \times 10^{-4}$ | **$1.4 \times 10^{-5}$ – $3.3\times 10^{-4}$** |
| CdS NCs in chloroform (4.6 nm size) [54] | 900 - 1000 nm; 100fs; 80 MHz | ~1.5% | ~$8.0 \times 10^{-6}$ – $1.5\times 10^{-4}$ | **~$1.2 \times 10^{-7}$ – $2.3 \times 10^{-6}$** |
| CdSe/CdS NCs in toluene (5 nm size) [39] | 1300 nm; 100fs; 1 kHz | N.A. | $4.3 \times 10^{-4}$ | **< $4.3 \times 10^{-4}$** |
| CdSe/CdS/ZnS NCs in toluene (6.6 nm size) [39] | 1300 nm; 100fs; 1 kHz | N.A. | $2.8 \times 10^{-3}$ | **< $2.8 \times 10^{-3}$** |
| ZnSe/ZnS NCs in water (4.4 nm) [55] | 700 & 800 nm; 200 fs; 1kHz | ~ 1.5% | $3.1 \times 10^{-4}$ – $1.9 \times 10^{-3}$ | **~$4.7\times 10^{-6}$– $2.9 \times10^{-5}$** |
| ZnS NCs in aqueous solution (2.5 nm) [56] | 780 nm; 120 fs; 1kHz | N.A. | $2.7 \times 10^{-4}$ | **< $2.7\times 10^{-4}$** |
| ZnSe NCs in butanol (3.5 nm & 4.5 nm) [7,8] | 1064 nm; 35 ps; 10Hz | ~ 2% | $1.2 \times 10^{-1}$ – $2.0 \times 10^{-1}$ | **$2.4\times 10^{-3}$ – $4.0 \times 10^{-3}$** |
| ZnSe/ZnS NCs in *n*-butanol (3.3 & 4.3 nm) [27,57] | 1064 nm; 35 ps; 10Hz | 17% & 22% | $1.4 \times 10^{-1}$ – $2.4 \times 10^{-1}$ | **$3.1 \times 10^{-2}$ – $4.1 \times 10^{-2}$** |
| **The applied excitation laser intensity generate electron-hole pairs per NC much greater than 1 and three-photon-excited intraband absorption should be significant as revealed by [58]** | | | | |
| **Large size CdSe/CdS dot in rod hetero-structure (2.4 nm size CdSe NC; 5 nm diameter, 24 - 39 nm length CdS nanorod) [59,60]** | 1300 nm; 150 fs; 1 kHz | 32% - 61% | $5.9 \times 10^{-2}$ – $1.5 \times 10^{-1}$ | **$1.9 \times 10^{-2}$ – $8.4 \times 10^{-2}$** |

| | | | | |
|---|---|---|---|---|
| **Organic molecules** | | | | |
| Fluorene-containing ferrocene derivatives[61] | 1260-1600 nm; 160 fs; 1kHz | N.A. | $4.6 \times 10^{-7} – 8.3 \times 10^{-7}$ | **$< 4.6 \times 10^{-7} – 8.3 \times 10^{-7}$** |
| Ri(9,9-diethyl-9 H-fluorenyl)amine [62] | 1160-1380 nm; 100 fs; 1kHz | 40% | $\sim 2.1 \times 10^{-7} – 4.0 \times 10^{-5}$ | **$\sim 8.4 \times 10^{-8} – 1.6 \times 10^{-5}$** |
| (E)-3-(4-(2-(1-hexyl-4-methyl-1H-imidazol-5-yl)vinyl)pyridinium-1-yl)propyl sulphate (IPPS) [46] | 2100 nm; 120 fs; 1kHz | ~ 4.4% | $3.7 \times 10^{-6}$ | **$\sim 1.6 \times 10^{-7}$** |
| Highly extended diphenylamino-end-capped ladder-type oligo-(p-phenylene)s [35] | 1230-1330 nm; 120 fs; 1kHz | 84% - 90% | $2.0 \times 10^{-3} – 4.6 \times 10^{-2}$ | **$1.7 \times 10^{-3} – 4.1 \times 10^{-2}$** |
| **Metal complexes** | | | | |
| Zn(II) -coordinated 3D MOFs with trans,trans -9, 10-bis (4-pyridylethenyl) anthracene (An2Py) ligand [51] | 950 – 1400 nm; 150 fs; 1kHz | 17% - 26% | $\sim 2.4 \times 10^{-7} – 1.5 \times 10^{-4}$ | **$\sim 4.0 \times 10^{-8} – 3.9 \times 10^{-5}$** |

**Supplementary Table 4.** Comparison of the *four-photon action cross-sections* of the $MAPbBr_3$, $MAPbBr_3/(OA)_2PbBr_4$, $CsPbBr_3$ NCs with the only few reported values from organic molecules and metal-organic framework.

| **Materials** | **Excitation laser source** | **PLQY** | **$\sigma_4$ ($10^{-104}cm^8s^3$ /photon$^3$)** | **$\eta\sigma_4$ ($10^{-104}cm^{10}s^4$ /photon$^4$)** |
|---|---|---|---|---|
| $MAPbBr_3$ NCs in toluene (8-9 nm size, sphere) (our result) | 1550 - 2000 nm; 50 fs; 1kHz | ~84% | $4.2 \times 10^{-2} – 3.6$ | **$\sim 3.6 \times 10^{-2} – 3.0$** |
| $MAPbBr_3/(OA)_2PbBr_4$ NCs in toluene (9-10 nm size, sphere) (our result) | 1550- 2000 nm; 50 fs; 1kHz | ~92% | $2.3 \times 10^{-1} – 2.6 \times 10^1$ | **$\sim 2.1 \times 10^{-1} – 2.4 \times 10^1$** |
| $CsPbBr_3$ NCs in toluene (~ 9 nm size, cube) (our result) | 1550-2000 nm; 50 fs; 1kHz | ~55% | $1.3 \times 10^{-1} – 1.3 \times 10^1$ | **$\sim 7.1 \times 10^{-2} – 6.9$** |
| **Organic molecules** | | | | |
| Stilbazo-lium dye compounds (PRL-L3) [47] | 1890 nm; 160 fs; 1kHz | N.A. | $1.4 \times 10^{-7}$ | **$< 1.4 \times 10^{-7}$** |
| (7-benzothiazol-2-yl-9,-di decylfluoren-2-yl)-Diphenylamine (NPH2Bz) [48,49] | 1600 nm; 35 ps; 10Hz | 70% | $8.1 \times 10^{-5}$ | **$5.6 \times 10^{-5}$** |
| **Conjugated Polymers** | | | | |
| Poly(2-methoxy-5-(2′-ethylhexyloxy)-1,4-phenylenevinylene) (MEH-PPV) [50] | 1780 - 1900 nm; 150 fs; 1kHz | | $\sim 2.3 \times 10^{-5} – 6.9 \times 10^{-5}$ | **$< 2.3 \times 10^{-5} – 6.9 \times 10^{-5}$** |
| **Metal complexes** | | | | |
| Zn(II) -coordinated 3D MOFs with trans,trans -9, 10-bis (4-pyridylethenyl) anthracene (An2Py) ligand [51] | 1450 – 1500 nm; 150 fs; 1kHz | 17% - 26% | $\sim 1.8 \times 10^{-6} – 2.1 \times 10^{-5}$ | **$\sim 3.0 \times 10^{-7} – 5.5 \times 10^{-6}$** |

**Supplementary Table 5.** Comparison of the *five-photon action cross-sections* of the $MAPbBr_3$, $MAPbBr_3/(OA)_2PbBr_4$, $CsPbBr_3$ NCs with the only two reported values from organic molecules.

| **Materials** | **Excitation laser source** | **PLQY** | **$\sigma_5$ ($10^{-136}cm^{10}s^4$ /photon$^4$)** | **$\eta\sigma_5$ ($10^{-136}cm^{10}s^4$ /photon$^4$)** |
|---|---|---|---|---|
| $MAPbBr_3$ NCs in toluene (8-9 nm size, sphere) (our result) | 2050 - 2300 nm; 50 fs; 1kHz | ~84% | $4.6 \times 10^{-2} – 2.9$ | **~$3.9 \times 10^{-2} – 2.4$** |
| $MAPbBr_3/(OA)_2PbBr_4$ NCs in toluene (9-10 nm size, sphere) (our result) | 2050 - 2300 nm; 50 fs; 1kHz | ~92% | $3.1 \times 10^{-1} – 2.2 \times 10^{1}$ | **~$2.9\times 10^{-1} – 2.0 \times 10^{1}$** |
| $CsPbBr_3$ NCs in toluene (~ 9 nm size, cube) (our result) | 2050-2300 nm; 50 fs; 1kHz | ~55% | $1.7 \times 10^{-1} – 1.2 \times 10^{1}$ | **~$9.2\times 10^{-2} – 6.5$** |
| **Organic molecules** | | | | |
| (E)-3-(4-(2-(1-hexyl-4-methyl-1H-imidazol-5-yl)vinyl)pyridinium-1-yl)propyl sulphate (IPPS) [46] | 2100 nm; 120 fs; 1kHz | ~ 4.4% | $1.9 \times 10^{-7}$ | **~$8.4 \times 10^{-9}$** |
| Highly extended diphenylamino-end-capped ladder-type oligo-(p-phenylene)s [35] | 2000 nm; 120 fs; 1kHz | 84% - 90% | Not quantified. But their $\sigma_{3max}$ are 4 orders larger than [29] and 2 - 3 orders smaller than our results. | **Not quantified. But their $\eta\sigma_{3max}$ are 4-5 orders larger than [29] and 2 - 3 orders smaller than our results.** |

**Supplementary Table 6.** SSA measurements to determine the pulse-width of the original 800nm laser pulse from the Libra Ti:Sapphire Amplifier System.

| | |
|---|---|
| O-scope delta (mS) | 1.600 |
| Mic Position 1 (mm) | 12.950 |
| Mic Position 2 (mm) | 12.880 |
| FWHM of SSA Pulse Trace (mS) | 0.240 |
| Calibration Value (pS/mS) | 0.292 |
| Autocorrelated PW (pS) | 0.070 |
| Gaussian PW (pS) | 0.050 |

## Supplementary Methods

### Absorption and photoluminescence (PL) by one-photon excitation

The one-photon absorption (1PA) and one-photon-excited PL spectra of the $MAPbBr_3$, $MAPbBr_3/(OA)_2PbBr_4$ and $CsPbBr_3$ NCs in toluene were measured using a Shimadzu UV1800 UV-VIS spectrophotometer and a Shimadzu RF-5301pc spectrofluorophotometer, respectively, as shown in Supplementary Figs 1a-c.The $CsPbBr_3$ NCs (of side ~9 nm) in toluene exhibit an absorption peak at ~505 nm and an excitonic PL peak at ~513.5 nm with narrow full-width at half maximum (FWHM) ~ 21 nm, consistent with reported values[1,2]. The 1PA peaks of $MAPbBr_3$ (diameter ~8-9 nm) and $MAPbBr_3/(OA)_2PbBr_4$ NCs (diameter ~9-10 nm) are located at ~510 nm & ~511 nm, and their excitonic PL peaks are at ~521 nm & ~520 nm with narrow FWHM of ~22.5 nm & ~24.5 nm, respectively, close to our previous report[3]. $MAPbBr_3/(OA)_2PbBr_4$ NCs possessed similar 1PA and excitonic PL spectra to its core-only counterpart. The 1PA peaks of $MAPbBr_3$, $MAPbBr_3/(OA)_2PbBr_4$ and $CsPbBr_3$ NCs exhibit clear blue shifts of ~15 nm and ~45 nm compared to their bulk counterparts[4-6], evident of the quantum confinement effect. The larger blue shift in $CsPbBr_3$ NC stems from its larger Bohr diameter ($d_B$ ~ 7 nm)[2] compared to $MAPbBr_3$ NC ($d_B$ ~ 4 nm)[7]. The relatively stronger quantum confinement in $CsPbBr_3$ NCs (*i.e.*, $d_B$ ~ 7nm comparable to 9 nm) gives rise to their larger MPA cross-sections than $MAPbBr_3$ NCs (*i.e.*, weak confinement $d_B$ ~ 4 nm << 8-9 nm) as discussed in the main manuscript. The 1PA spectra of the NCs presented in Supplementary Figs 1a-calso revealed that they exhibit negligible 1PA at wavelengths longer than ~ 530 nm. Supplementary Figure 1d shows the 1PA spectra and one-photon excited PL spectra of the $(OA)_2PbBr_4$ thin film, in which the excitonic 1PA peak at ~ 393 nm is revealed and the PL peak is located at ~ 402 nm.

The absolute photoluminescence quantum yields (PLQYs) of the perovskite NCs were measured using an Ocean-optics USB4000 spectrometer with a $BaSO_4$-coated integrating sphere excited by a laser beam at 400 nm. The PLQY of $MAPbBr_3$ NCs was measured to be ~84%; while the PLQY of the core-shell

$MAPbBr_3/(OA)_2PbBr_4$ NCs was ~92%. Its increased PLQY and the enhanced stability (retaining a 60% PLQY after two months) is attributed to the effective surface passivation and efficient carrier confinement provided by the shell, as detailed in Ref [3]. The PLQY of $CsPbBr_3$ NCs was determined to be ~ 55%, which is close to the reported value in the literature[2].

**Structural characterization**

Representative transmission electron microscopy (TEM) images of the as-synthesized NCs recorded by Jeol JEM-2010 are displayed in the insets of Supplementary Figs 1a-c, to illustrate their shapes and sizes. As shown, the cubic $CsPbBr_3$ NCs with size ~9 nm is comparable to recent reports[1,2]. In addition, both the core-only $MAPbBr_3$ NCs and the core-shell $MAPbBr_3/(OA)_2PbBr_4$ NCs possess spherical shape, with sizes of approximately 8-9 nm and 9-10 nm, respectively. Supplementary Figure 1eshows the X-ray diffraction (XRD) patterns of the $MAPbBr_3$, $MAPbBr_3/(OA)_2PbBr_4$ and $CsPbBr_3$ NCs acquired by XRD Bruker D8 Advance, for the structural characterization of the NCs. Generally, the organic-inorganic perovskite structure $AMX_3$ (A = $CH_3NH_3$, $CH(NH_2)_2$, Cs; X= I, Br, Cl; M= Pb, Sn) is formed by a three-dimensional (3D) network with small organic or inorganic cations occupying the A-site, and divalent metal (M) and halides (X) forming the $MX_6$ octahedral. The XRD peak positions for the $MAPbBr_3$ NCs and $CsPbBr_3$ NCs (Supplementary Fig. 1e) match well with the cubic structure of reported patterns for the bulk system[5], with a *d*-spacing of around 5.9 Å. However, if larger cations (>2.6 Å) are introduced[8], the cubic symmetry is broken. Instead, layered structures of general formula $(RNH_3)_2A_{n-1}M_nX_{3n+1}$ ($n$ = 1, pure 2D layered; $n = \infty$, pure 3D structure; and $n$ = defined integer, quasi-2D layered structure) are formed. The diffraction pattern of $MAPbBr_3/(OA)_2PbBr_4$ NCs (Supplementary Fig. 1e) show additional peaks at lower diffraction angles, originating for layered structures ($(OA)_2PbBr_4$), along with peaks associated with 3D perovskite formation. Although the (100) and (200) peak intensity is lower for the $MAPbBr_3/(OA)_2PbBr_4$ NCs compared to $MAPbBr_3$ NCs, the relative intensity difference and peak positions remains unchanged.

**Photo electron spectroscopy in air (PESA) measurements for $MAPbBr_3$ and $(OA)_2PbBr_4$ thin films**

Photo electron spectroscopy in air (PESA) measurements on $MAPbBr_3$ and $(OA)_2PbBr_4$ thin films were performed to estimate the valance band maximum (VBM) alignment between the core and shell in the core-shell $MAPbBr_3/(OA)_2PbBr_4$ NCs, and to avoid the influence from the organic ligands on surface of NCs films. A Riken Keiki AC-2 PESA spectrometer was utilized to perform the PESA measurements with a power setting of 800 nW and a power number of 0.5 as shown in Supplementary Fig. 1f, the PESA results of $MAPbBr_3$ and $(OA)_2PbBr_4$ thin films reveals their VBM at -5.62 eV and -6.00 eV, respectively. Together with the bandgap values determined from the 1PA spectra in Supplementary Fig. 1and in the reference[4-5], a type-I conduction and valence band-edge alignment between the core and shell in the core-shell $MAPbBr_3/(OA)_2PbBr_4$ NCs is expected, as shown in Fig. 1ain the main text.

**Theoretical calculations to determine the conduction and valence band-edge alignment in core-shell perovskite NCs**

We employed the all-electron-like projector augmented wave (PAW) method[9] and the Perdew-Burke-Ernserhof (PBE) exchange correlation potential[10] as implemented in the VASP code[11]. In the basis, the semicores of Ti and Pb atoms were treated as valence electrons, *i.e.*, 14 valence electrons for Pb ($5d^{10}6s^26p^2$) atom. The 2×2×1 Monkhorst-Pack grid of k-points[12] for Brillouin zone integration of $MAPbBr_3/(OA)_2PbBr_4$, was used in the calculations of structure relaxation. The cut-off energy for the plane wave expansion of the wave functions was 400eV. All atoms were fully relaxed.

For the band offset of $MAPbBr_3/(OA)_2PbBr_4$ calculations, we first performed separate calculations for the band offsets of bulk $MAPbBr_3$ and $(OA)_2PbBr_4$ to determine the VBM ($E_{\mathrm{VBM}}^{\alpha}$, $\alpha =$ $MAPbBr_3$, $(OA)_2PbBr_4$) with respect to their atomic 1s-core energy level ($E_{1s}^{\alpha}$) in the bulk solid. Secondly, we also performed the self-consistent calculations of $MAPbBr_3/(OA)_2PbBr_4$ superlattice in order to align the atomic 1s-core energy level of $MAPbBr_3$ and $(OA)_2PbBr_4$. Their atomic 1s-core energy levels ($E_{1s}^{\mathrm{if}}$) were taken in

the bulk-like regions of both sides, which were far from the interface zone. Thus, the band offset of $MAPbBr_3/(OA)_2PbBr_4$ is given by:

$$\Delta E_{\mathrm{VBM}} = \left(E_{\mathrm{VBM}}^{\mathrm{bulk}}(\mathrm{MAPbBr_3}) - E_{\mathrm{1s}}^{\mathrm{bulk}}(\mathrm{MAPbBr_3})\right) - \left(E_{\mathrm{VBM}}^{\mathrm{bulk}}((\mathrm{OA})_2\mathrm{PbBr_4}) - E_{\mathrm{1s}}^{\mathrm{bulk}}((\mathrm{OA})_2\mathrm{PbBr_4})\right) + \left(E_{\mathrm{VBM}}^{\mathrm{if}}(\mathrm{MAPbBr_3}) - E_{\mathrm{VBM}}^{\mathrm{if}}((\mathrm{OA})_2\mathrm{PbBr_4})\right) \quad \text{(Supplementary equation 1)}$$

The detailed calculation method can be found in ref. 13. In Supplementary Fig. 2, we present the calculated band offset of $MAPbBr_3/(OA)_2PbBr_4$, where the band gaps are experimental values. We find that the VBM of $MAPbBr_3$ is 0.39 eV higher than $(OA)_2PbBr_4$, which shows good agreement with the experimental result (0.38 eV). Our theoretical results also revealed a type I band offset.

## **Femtosecond laser specifications from manufacturer's data sheets**

The excitation laser pulses applied in our measurements were generated by an optical parametric amplifier (OPA) (OperA-Solo, Coherent) pumped by a regenerative amplified femtosecond Ti:Sapphire laser system (~50 fs, 800 nm, 1 kHz,; Libra, Coherent). The Coherent Libra regenerative amplifier was seeded by a femtosecond Ti:Sapphire oscillator (~50 fs, 80 MHz, Vitesse, Coherent).

Based on the specification data sheet for Coherent, Libra™ Ti:Sapphire Amplifier System, the original 800nm laser pulses follow the Gaussian distribution and have pulse-width (FWHM) about 50 fs (https://www.coherent.com/lasers/laser/libra-series). In addition, based on the specification data sheet of the Coherent, OperA-Solo optical parametric amplifier (OPA), the output laser pulses (1 kHz, 250-2600 nm) from the OPA with about 50fs, 800nm laser pulses for pumping possess pulse-widths about (1 to 1.2) times of the pump laser pulse duration (*i.e.*, about 50 – 60 fs) and follow the Gaussian distribution (https://www.coherent.com/lasers/laser/opera-solo-ultrafast-optical-parametric-amplifier). Hence, the laser pulses applied in our measurements (675nm – 2300nm from OperA-Solo OPA and 800nm from Libra Ti:Sapphire Amplifier) should possess pulse widths in the range of about 50 – 60 fs with maximum variation

about 20%. Therefore, the variation in the pulse-widths at different wavelengths will not lead to variation of the order of the measured 5PA cross-sections in the lead bromide perovskite NCs.

**Temporal distribution and pulse width characterization for laser beam at 800 nm utilizing Single-Shot Autocorrelator (SSA)**

A Single-Shot Autocorrelator (SSA) (High Resolution SSA, Coherent) was used to measure the pulse-width of the 800 nm output laser pulses from Libra Ti:Sapphire Amplifier. Supplementary Table 6 summarizes the obtained results. An autocorrelated pulse-width of the original 800 nm laser pulses could be acquired from the product between the calibration value and the time width of the autocorrelation signal on the oscilloscope (FWHM), which was about 70 fs as shown in the below table S1. Since the laser pulse follows the Gaussian distribution, a constant factor 0.707 was multiplied to obtain the pulse-width of the original 800 nm laser pulses, which is about 50fs. The measured pulse-width of the original 800 nm laser pulses is in good agreement with the specifications provided on the data sheet of the Coherent, Libra Ti:Sapphire Amplifier System.

**Direct characterization of the temporal and spectral distributions of the applied femtosecond laser beams at 700-1100 nm utilizing frequency-resolved optical gating (FROG)**

Since our SSA is only suitable to measure the pulse-width of laser pulses at 800nm, we have further utilized frequency-resolved optical gating (FROG) (Swamp Optics, UPM-8-50) to characterize the pulse-widths of the laser pulses from both the Libra Ti:Sapphire Amplifier (original 800 nm laser beam) and the OperA-Solo OPA in the wavelength range 700 – 1100nm. The characterization wavelength range is limited by the capability of our FROG set-up. Figure S3 shows the acquired two-dimensional FROG traces, temporal distributions and spectral distributions of the laser pulses in the wavelength range 700 – 1100nm. Supplementary Figures 15a, d, g, j, m, p, s, v, y displayed the measured two-dimensional FROG traces of the

laser pulses, Supplementary Figs 15b, e, h, k, n, q, t, w, z demonstrated the corresponding temporal distributions and Supplementary Figs 15c, f, i, l, o, r, x, aa displayed the corresponding spectral distributions. The laser pulses at 800 nm was from the Libra Ti:Sapphire Amplifier, whereas the laser pulses at other wavelengths were generated by the OperA-Solo OPA.

As shown in Supplementary Fig. 15, both the temporal and the spectral distributions of the laser pulses in the wavelength range 700 – 1100nm can be well-fitted by the Gaussian function, indicating laser pulses in the wavelength range indeed follow the Gaussian temporal distribution. The measured pulse-width of the original 800 nm laser pulses is about 50.6 ± 5.1 fs, which agrees well with the result acquired from SSA and the specification of the Coherent, Libra Ti:Sapphire Amplifier System (https://www.coherent.com/lasers/laser/libra-series). Moreover, as summarized in Figure S3ad, the measured pulse-widths of the laser pulses in wavelength range 700 – 1100nm range from 50.6 ± 5.1 fs to 56.3 ± 5.6 fs with variation within 15%. The acquired pulse-width result is in good agreement with the specification of OperA-Solo OPA (https://www.coherent.com/lasers/laser/opera-solo-ultrafast-optical-parametric-amplifier). Hence, the variation in the pulse-widths at different wavelengths in the range 700 – 1100nm will not lead to variation of the order of the measured 5PA cross-sections in the lead bromide perovskite NCs.

In addition, Supplementary Fig. 15ae demonstrated that the measured spectral bandwidths and temporal pulse-widths followed the time-bandwidth relation of Fourier-transform-limited pulses ($\Delta\lambda_{FWHM} = (A \times K / c) \times ((\lambda_0)^2 / \Delta\tau_{FWHM})$)[14], a typical characteristic of ultrashort laser pulses generated by mode-locked lasers. $c$ is the speed of light in vacuum, $K$ is a constant depending only on the pulse shape and equals to 0.441 for Gaussian temporal distribution, $A$ is a scaling factor called time-transform-limit factor. From the best fit to the experimental results, $A$ was determined to be about 1.27, as indicated in Supplementary Fig. 15ae.

**Direct characterization of the spectral distributions and indirect characterization of the temporal distributions of the applied femtosecond laser beams at 675 – 1700 nm utilizing a visible monochromator coupled with a CCD and an infrared monochromator coupled with a liquid-nitrogen-cooled InGaAs infrared detector**

Unfortunately, due to limited available wavelength range of our FROG set-up, we could not perform direct measurements on the pulse-widths of utilized laser pulses at wavelengths beyond the range of 700 – 1100nm (*i.e.*, 675 and 1200-2300 nm). Based on the above verified pulse-width-bandwidth relation of Fourier-transform-limited pulses ($\Delta\lambda_{FWHM} = (A \times K / c) \times ((\lambda_0)^2 / \Delta\tau_{FWHM})$)[14], we have measured the spectral profiles of laser pulses at wavelength range 675 – 1700 nm to indirectly derive the pulse-widths at these wavelengths. The overlapping wavelength range of 700 – 1100 nm using this approach serves as a self-consistent cross-check with the results obtained from the FROG approach. The visible monochromator (Acton, Spectra Pro 2750i) coupled with CCD (Princeton Instruments, Pixis 100B) and infrared monochromator (Acton, Spectra Pro 2300i) coupled with liquid-nitrogen-cooled InGaAs infrared detector (Princeton Instruments, 7490-0001) were employed to acquire the optical spectra of the laser beam. The upper-limit of the characterized wavelength range is determined by the capability of our infrared monochromator (Acton, Spectra Pro 2300i) coupled with liquid-nitrogen-cooled InGaAs infrared detector (Princeton Instruments, 7490-0001).

The acquired optical spectra of the laser pulses at wavelength range 675 – 1700 nm were displayed in Supplementary Fig. 16. Supplementary Figs 16a-q displayed the measured optical spectra at the wavelengths range from 675 – 1700 nm, respectively. The negligible *Ch*^2 and the very close to unity *R*^2 at all wavelengths summarized in Supplementary Fig. 16r demonstrates the good Gaussian fitting, revealing the Gaussian spectral distribution of the laser pulses at these wavelengths. This is highly consistent with the results acquired from FROG measurements and indicates Gaussian temporal distributions at the wavelength range 675 – 1700 nm, beyond the one obtained from FROG results. Due to the limitation of our

experimental set-up, we could not directly measure the optical spectra at wavelength range 1800 – 2300 nm. However, since the laser pulses at wavelength range 1700 – 2300 nm belong to the same idler part of the OperA Solo output, laser pulses at wavelength range 1800 – 2300 nm are expected to possess similar Gaussian temporal and spectral distributions as at 1700 nm.

Supplementary Figure 16s displayed the comparison between the acquired spectral bandwidths at 700 – 1100 nm by spectrometer and that obtained from the FROG measurements. The acquired spectral bandwidths at different wavelengths were summarized in Supplementary Fig. 16t. The spectral bandwidths obtained at wavelength range 700 – 1000 nm are in excellent agreement with the ones measured by FROG, with variation less than 5%. Moreover, the derived pulse-widths at wavelengths of 675 & 1200 - 1700 nm based on the verified pulse-width-bandwidth relation of Fourier-transform-limited pulses and its comparison with the measured pulse-widths at 700 – 1100 nm were shown in Supplementary Fig. 16t. The pulse widths at wavelength range 675 – 1700 nm are in the range of (50.6 ± 5.1) – (62.9 ± 6.3) fs, with maximum variation about 25%, consistent with the specifications of the OperA-Solo OPA. The measured pulse-widths are slightly stretched at longer wavelengths (maximum around 63 fs) compared to the value we had used in original calculations (50 fs). This will lead to an overestimation of the applied laser peak intensities and consequently an underestimation of the multi-photon absorption cross-sections at longer wavelengths for our reported values. For example, let us consider that the actual pulse widths are in fact longer at 63 fs for five-photon excitations instead of 50 fs used in the manuscript (*i.e.*, a pulse width stretch of about 25%) Based on the expression of the five-photon excited upconversion fluorescence of $(\eta\sigma_5) \propto F_5/(\tau_{\mathrm{FWHM}} I_0^5) \propto 1/(\tau_{\mathrm{FWHM}} \cdot 1/(\tau_{\mathrm{FWHM}})^5) \propto (\tau_{\mathrm{FWHM}})^4$ (Refs. [15, 16] and Supplementary equation 8, $\tau_{\mathrm{FWHM}}$ is the pulse width, $I_0$ is the laser peak intensity and $F_5$ is the five-photon excited upconversion PL strength), the five-photon action cross-section ($\eta\sigma_5$) and 5PA cross-sections ($\sigma_5$) would be in fact larger than our reported value (*i.e.*, about 2.5 times larger). Therefore, our reported 5PA cross-sections are conservative values.

Measurements of the spectral profile at wavelengths in the range 1800 – 2300 nm are beyond the capability of our experimental set-up. However, as mentioned, laser beams at wavelength range 1700 – 2300 nm belong to the same idler part of the OperA Solo OPA output. Therefore, laser pulses at wavelength range 1800 – 2300 nm are expected to have similar or slightly more stretched pulse duration, which is also in agreement with the specification of the OperA-Solo OPA that the output laser pulses (1 kHz, 250-2600 nm) from it (with about 50fs, 800nm laser pulses pumping) possess pulse-widths about (1 - 1.2) times of the pump laser pulse duration (*i.e.*, about 50 – 60 fs).

In summary, we have carefully rechecked all the temporal and spectral uncertainties of the applied laser sources highlighted by the reviewer. The applied laser pulses at wavelengths 675 – 2300 nm were demonstrated to possess Gaussian temporal and spectral distribution. And the laser pulses at different wavelengths have similar pulse durations with maximum variation about 25%. The original laser pulse at 800 nm output from the Libra Ti:Sapphire Amplifier possess the minimum pulse duration of (50.6 ± 5.1) fs, and the laser pulses output from OperA-Solo OPA are slightly stretched. In addition, the laser pulses at longer wavelengths were found to have slightly more stretched pulse duration, which will lead to overestimation of the applied laser peak intensities and consequently an underestimation of the multi-photon absorption cross-sections at longer wavelengths in our reported values. Nonetheless, such underestimation will not change the order of the measured 5PA cross-sections. Our reported 5PA cross-sections are in fact conservative values.

**Characterization of the spatial distributions of the applied femtosecond laser beams at various wavelengths using knife-edge scan method**

Knife-edge beam scans[17,18] was performed along both the $x$- and $y$-directions of the cross-sectional plane of the laser beam at different wavelengths to characterize its spatial profile. In the measurements, as the knife-edge was moved along both the $x$- and $y$-directions of cross-section plane of laser beams, a power/energy meter (for measuring the average power or pulse energy of ultrashort laser pulses - Coherent, LabMax-Top) was applied to measure the total transmitted energy as a function of the scan distance along the $x$- or $y$-direction (*i.e.*, $x$- or $y$-position).

Supplementary Fig. 17 shows the acquired knife-edge scan curves along both the $x$- and $y$-direction at wavelengths in the range 675-2300 nm. The measured normalized knife-edge scan curves along both the $x$- and $y$-direction at all wavelengths is displayed in Supplementary Figs 17a-x, together with the Gaussian fitting[17-19]. The acquired energy at each point ($x$ or $y$) is the average of ten measured values with a time interval of 0.5s. The expression for the Gaussian fitting is: $E(x)/E_{max} = E_0/E_{max} + 1/2(1 + erf(\sqrt{2}(x - x_0)/w))$, similar expression also applies to the $y$-direction by simply exchanging $x$ with $y$[17-19]. $E(x)$ is measured laser energy at scan distance $x$, $E_{max}$ is the maximum energy and $E_0$ is background energy, $x_0$ is a position of shift with the half of the real energy, $erf$ is a standard error function and $w$ is the measured laser beam radius ($1/e^2$ radius). The acquired knife-edge scan curves in both $x$- and $y$-directions at all wavelengths were well-fitted using the above Gaussian expression, as shown in Supplementary Figs 17a-x. Supplementary Figs 17y, z summarized the negligible $Ch$^2 and the very close to unity $R$^2 for the Gaussian fitting at all wavelengths in both the $x$- and $y$-direction, indicating excellent Gaussian fitting and consequently the Gaussian spatial distribution of laser beams along both directions at all wavelengths. Moreover, the high consistency between the measured knife-edge scans in the $x$ direction and $y$ direction at all wavelengths manifested in Supplementary Figs 17a-x reveals the two-dimension Gaussian spatial distribution of the laser beams in the cross-section plane. Supplementary Fig. 17aa summarized the acquired

laser beam radiuses along both *x* and *y* directions at all wavelengths. The measured laser beam radii in *x*-direction at all wavelengths are in good agreement with the ones measured by in *y*-direction with variation less than 6 %, further demonstrating that the two-dimension spatial profiles of the laser beams in the cross-sectional plane follow the Gaussian distribution. Hence, the knife edge beam scans along both the *x*- and *y*-directions of cross-sectional plane of laser beams at different wavelengths validate that spatial profiles of the applied laser beams followed the Gaussian distribution.

In addition, as in Supplementary Fig. 17aa, the laser beam radius at all wavelengths are in range of (1.8 ± 0.2) – (4.1 ± 0.4) mm (*i.e.*, laser beam diameters (3.6 ± 0.4) – (8.2 ± 0.8) mm). Laser beam output from the Libra Ti:Sapphire Amplifier at 800 nm was found to have the largest beam radius of (4.1 ± 0.4) mm. Within each different laser output component from the OperA Solo OPA (*i.e.*, second harmonic signal, second harmonic idler, single and idler), the acquired beam radius increases with increasing wavelengths. In addition, for all the laser beams from the OperA Solo OPA, the acquired beam radii followed the overall increasing trend with increasing wavelengths.

In summary, we have carefully rechecked the spatial uncertainties of the applied laser sources highlighted by the reviewer. Through the knife-edge beam scans along both the *x*-and *y*-directions of cross-sectional plane of laser beams, the spatial profiles of the applied laser beams at wavelengths in the range 675-2300 nm were validated to follow the Gaussian distribution. The measured laser beam radii at all wavelengths are in range of (1.8 ± 0.2) – (4.1 ± 0.4) mm. The maximum beam radius was at 800 nm from the Libra Ti:Sapphire Amplifier and beam radii of the laser beams from OperA Solo OPA follow an overall increasing trend with increasing wavelengths.

## Supplementary Notes

### Open-aperture *Z*-scan measurements for quantifying 2PA cross-sections ($\sigma_2$) at 800 nm and 3PA cross-sections ($\sigma_3$) at 1050 and 1100 nm

Open-aperture *Z*-scan measurements[20] were conducted to quantify the $\sigma_2$ values (at 800 nm) of $MAPbBr_3$, $MAPbBr_3/(OA)_2PbBr_4$ and $CsPbBr_3$ NCs, which were dispersed in toluene solvent and contained in 1-mm-thick quartz cuvettes. In open-aperture *Z*-scans, the excitation laser pulses (~ 50 fs, 800 nm, 1 kHz) were generated by the regenerative amplified femtosecond Ti:Sapphire laser system (Libra, Coherent), which was seeded by a femtosecond Ti:Sapphire oscillator (~ 50 fs, 80 MHz, Vitesse, Coherent). A schematic illustration of the open-aperture *Z*-scan set-up is shown in Supplementary Fig. 3. A beam splitter was employed to divide the incident laser beam into two parts. The first part served as the reference and was directed into a reference power detector ($D_R$). The other part functioned as the signal beam and was focused by a circular lens with 20 cm focus length onto a 1-mm-thick quartz cuvette filled with the toluene solution of perovskite NCs. The transmitted signal beam through NCs was detected by a signal power detector ($D_S$). The sample transverses back and forth along the propagation direction of the laser beam (*z*-axis) on a linear motorized stage. The transmission of the sample ($D_S/D_R$) was monitored while translating the sample through the focal point, and the transmission was recorded as a function of the sample position ($z$). With the incident laser pulse energies kept at a constant level, the sample experiences various laser irradiance $I(z)$ at different *z*-positions, giving rise to corresponding changes in transmission if the sample absorbs light nonlinearly.

A wide-gap semiconductor CdS was utilized as the standard sample for the calibration of our open-aperture *Z*-scan set-up. Supplementary Fig. 4a illustrates the measured open-aperture *Z*-scan curves on the 0.5-mm thick CdS wafer at 800 nm and under excitation peak intensities of ~1.0 and 1.9 GW $cm^{-2}$. The extracted 2PA coefficient $\beta$ ~ 5.9 cm $GW^{-1}$ is in good agreement with both theoretical prediction[21] and

experimental report[22], indicating that the set-up is properly calibrated. The open-aperture *Z*-scan responses of the toluene solutions of $MAPbBr_3$ (~ 2.0 μM), $MAPbBr_3/(OA)_2PbBr_4$ (~ 2.1 μM) and $CsPbBr_3$ NCs (~ 1.0 μM) in 1-mm-thick cuvette acquired at 800 nm with peak intensity of ~ 25.0 and ~ 35.0 GW $cm^{-2}$ are presented in Supplementary Figs 4b-d, respectively. Open-aperture *Z*-scan measurement on the pure toluene solvent was also conducted under the same excitation condition (~ 35.0 GW $cm^{-2}$ excitation at 800 nm) as the control experiment, as shown in Supplementary Fig. 4d. The almost flat open-aperture *Z*-scan curve of the toluene suggests its negligible nonlinear absorption response at the applied excitation condition.

2PA cross-sections of the NC solutions can be obtained through fitting the normalized transmittance to the well-established *Z*-scan theory[20] as:

$$T(z) = \frac{(1-R)^2}{\sqrt{\pi}q(z)} \int_{-\infty}^{\infty} ln\left[1 + q(z)e^{-x^2}\right]dx \qquad \text{(Supplementary equation 2)}$$

$q(z) = (1-R)\beta I_{00} L_{eff}/(1 + z^2/z_0^2), \beta = N\sigma_2/(\hbar\omega)$. $R$ is reflection from the sample front surface; $I_{00}$ is peak intensity at the focal point of the incident laser beam; $L_{eff} = L$ at 800 nm and $L$ is sample thickness; $N$ is concentration of NCs; $\hbar\omega$ is the incident photon energy and $\sigma_2$ is the 2PA cross-section. 2PA cross-sections ($\sigma_2$) of the $MAPbBr_3$, $MAPbBr_3/(OA)_2PbBr_4$ and $CsPbBr_3$ NCs at 800 nm were estimated to be ~ (0.8 ± 0.1) × $10^6$ GM, ~ (5.0 ± 0.8) × $10^6$ GM and ~ (2.0 ± 0.3) × $10^6$ GM, respectively. The derived $\sigma_2$ value of the $CsPbBr_3$ NCs at 800 nm is close to the measured result in a recent report[2] (Supplementary Table 1), indicating that the *Z*-scan measurements in current work were appropriately conducted. On the other hand, the estimated $\sigma_2$ value of the $CsPbBr_3$ NCs at 800 nm here is about one order larger than another published value[1] (Supplementary Table 1). A plausible origin of the difference in the obtained $\sigma_2$ values might be the sample diversity utilized in the different measurements, as illustrated in Ref. [2]. Taking account of both the volume and the local field effects, the elucidated $\sigma_2$ value of the $MAPbBr_3$ NCs, is about three-orders of magnitude larger than the result corresponding to the recently reported 2PA coefficient of

bulk $MAPbBr_3$ crystal[23] ($\sigma_2$ corresponding to the bulk $MAPbBr_3$ crystal is derived based on the formula in ref. [24], with $\varepsilon_0 = 2.38$ and $\varepsilon = 25.5$ are dielectric constants of toluene and $MAPbBr_3$[25], respectively.). Such large enhancement results from the quantum confinement effect, which has been well-documented in conventional semiconductor NCs[26,27]. Moreover, the shell coating with $(OA)_2PbBr_4$ has further enhanced the $\sigma_2$ of the $MAPbBr_3/(OA)_2PbBr_4$ NCs at 800 nm by ~6.5 times. The ratio between the acquired $\sigma_2$ values of $CsPbBr_3$, $MAPbBr_3$ and $MAPbBr_3/(OA)_2PbBr_4$ NCs at 800 nm is consistent with that obtained from the two-photon-excited PL measurements displayed in Supplementary Figure 6a, further confirming that the measurements have been properly carried out.

The applied experimental set-up for the open-aperture *Z*-scan measurements on 3PA in the lead bromide perovskite NCs at 1050 and 1100 nm is similar to the one used for 2PA at 800 nm. The calibration of our open-aperture *Z*-scan measurements for 3PA at 1050 and 1100nm was conducted utilizing a wide-gap semiconductor CdS (0.5-mm thick CdS wafer, single crystal). 3PA of CdS single crystal have been well studied both experimentally[28] and theoretically[29] in this wavelength range. Open-aperture *Z*-scan curves on the CdS wafer under excitation peak intensities of about 23 and 36 GW $cm^{-2}$ at 1050 and 1100nm are displayed in Supplementary Figs 8a and 9a. Based on the open-aperture *Z*-scan theory for 3PA[30], Ln(1-$T_{OA}$) (where $T_{OA}$ is the normalized transmittance along the *Z*-axis in the open-aperture *Z*-scan) was plotted as a function of Ln($I_0$) ($I_0$ is the maximum excitation intensity on the *Z*-axis and $I_0 = I_{00}/(1 + z^2/z_0^2)$) at both wavelengths to demonstrate the presence of 3PA, as shown in the insets of Supplementary Figs 8a and 9a. The linear fit with slopes of about 1.95 and 2.03 are indicative of the occurrence of 3PA processes at both wavelengths, which is as expected since the photon energies of the applied laser beams are between one third and half of the bandgap energy of CdS. The extracted 3PA coefficients $\gamma$(1050 nm) ~ (1.4 ± 0.2) $\times 10^{-2}$ $cm^3GW^{-2}$ and $\gamma$(1100 nm) ~ (1.3 ± 0.2) $\times 10^{-2}$ $cm^3GW^{-2}$ agree well with both the experimental report[28] and theoretical calculation[29], indicating that our *Z*-scan set-up is properly calibrated.

Supplementary Figures 8b,c show the open-aperture $Z$-scan responses of 3PA in the toluene solutions of $MAPbBr_3$ (~2.0 μM) and $CsPbBr_3$ NCs (~1.0 μM) in 1-mm-thick cuvette under excitation at 1050 nm with peak intensities of ~35.0 and ~50.0 GW $cm^{-2}$. On the other hand, open-aperture $Z$-scans of 3PA in the $MAPbBr_3/(OA)_2PbBr_4$ NCs (~2.1 μM) excited at 1050 nm with peak intensities of ~25.0 and ~35.0 GW $cm^{-2}$ are shown in Supplementary Fig. 8d. The insets of Supplementary Figs 8b-c show the corresponding plots of $\mathrm{Ln}(1–T_{OA})$ vs. $\mathrm{Ln}(I_0)$ together with the linear fits, manifesting the presence of 3PA. The acquired slopes are about 1.82, 1.85 and 1.83, indicating relatively larger deviation from 2 (*i.e.*, 15-20%). Such deviation at 1050 nm is in accordance with the obtained slopes from the excitation fluence dependence of multi-photon excited upconversion PL, as shown in Fig. 2a of the original manuscript. And the deviation may result from the NCs size inhomogeneity leading to an admixture of contributions from both the 2PA and 3PA processes at this boundary wavelength of 1050nm (lines 138-142 of original manuscript). However, since the deviation is less than 20%, 3PA process still dominates at this boundary wavelength.

For excitation at 1100nm, open-aperture $Z$-scan curves of 3PA in the $MAPbBr_3$ and $CsPbBr_3$ NCs in toluene solution under excitation peak intensities of ~75.0 and ~ 100.0 GW $cm^{-2}$ were also acquired and shown in Supplementary Figs 9b,c. On the other hand, Supplementary Fig. 9d shows the open-aperture $Z$-scan curves of 3PA in $MAPbBr_3/(OA)_2PbBr_4$ NCs in toluene solution with excitation wavelength at 1100nm and excitation peak intensities of ~ 50.0 and ~ 75.0 GW $cm^{-2}$. Corresponding plots of $\mathrm{Ln}(1–T_{OA})$ vs. $\mathrm{Ln}(I_0)$ and the linear fits are also added to the insets of Supplementary Figs 9b-d, respectively, to validate the occurrence of the 3PA processes. The slopes obtained at 1100nm are about 1.97, 2.01 and 2.03 for $MAPbBr_3$, $CsPbBr_3$ and $MAPbBr_3/(OA)_2PbBr_4$ NCs, respectively, clearly indicating the only 3PA process at 1100 nm excitation.

Open-aperture $Z$-scan measurements on the pure toluene solvent were also conducted under the maximum excitation peak intensities (*i.e.*, ~ 50.0 GW $cm^{-2}$ excitation at 1050 nm and ~ 100.0 GW $cm^{-2}$ excitation at 1100 nm) as the control experiment, which were shown in Supplementary Figs 8d and 9d.

Almost flat open-aperture *Z*-scan curves of the toluene at both wavelengths suggest its negligible nonlinear absorption response at the applied excitation conditions.

To acquire the 3PA cross-sections of the NC solutions, the below well-established *Z*-scan theory[31] were applied to fit the normalized open-aperture *Z*-scan transmittance (considering the Gaussian spatial and temporal distributions of utilized laser beam):

$$T(z) = \frac{1}{\sqrt{\pi}p_0(z)} \int_{-\infty}^{\infty} ln\left\{\left[1 + p_0^2(z)e^{-2x^2}\right]^{1/2} + p_0(z)e^{-x^2}\right\} dx \qquad \text{(Supplementary equation 3)}$$

$p_0(z) = \left(2\gamma(1-R)^2 L'_{eff} I_{00}^2/(1 + z^2/z_0^2)^2\right)^{1/2}, \gamma = N\sigma_3/(\hbar\omega)^2$. $R$ is reflection from the sample front surface; $I_{00}$ is peak intensity at the focal point of the incident laser beam; $L'_{eff} = L$ at 1050 and 1100 nm and $L$ is sample thickness due to the negligible one-photon absorption at these wavelengths; $N$ is concentration of NCs; $\hbar\omega$ is the incident photon energy and $\sigma_3$ is the 3PA cross-section. 3PA cross-sections ($\sigma_3$) of the $MAPbBr_3$, $MAPbBr_3/(OA)_2PbBr_4$ and $CsPbBr_3$ NCs at 1050 nm were estimated to be ~ (2.3 ± 0.3) × $10^{-74}$ $cm^6s^2photon^{-2}$, ~ (20 ± 3) ×$10^{-74}$ $cm^6s^2photon^{-2}$ and ~ (6.7± 1.0) × $10^{-74}$ $cm^6s^2photon^{-2}$, respectively. Moreover, for excitation at 1100 nm, the 3PA cross-sections ($\sigma_3$) of the $MAPbBr_3$, $MAPbBr_3/(OA)_2PbBr_4$ and $CsPbBr_3$ NCs were calculated to be ~ (0.55 ± 0.08) × $10^{-74}$ $cm^6s^2photon^{-2}$, ~ (3.3 ± 0.5) ×$10^{-74}$ $cm^6s^2photon^{-2}$ and ~ (1.9 ± 0.3) × $10^{-74}$ $cm^6s^2photon^{-2}$.

The obtained $\sigma_3$ of perovskite NCs through the open-aperture *Z*-scan measurements are highly consistent with the results acquired from multi-photon excited upconversion PL measurements (Fig. 2c and Table 1 of the original manuscript) with the variation in the range of about 6%-17%. Hence, the open-aperture *Z*-scan measurements on 3PA in the perovskite NCs performed at 1050 and 1100 nm further confirm our 3PA results measured with the multi-photon excited upconversion PL technique. The good agreement of the 2PA (as shown above and in Supplementary Figs 4, 6, 7) and 3PA (as in Supplementary Figs 6-9) cross-sections acquired by the multi-photon excited upconversion PL technique with that obtained from open-aperture

measurements validates that the multi-photon absorption cross-sections of our perovskite NCs have been properly measured with the multi-photon excited upconversion PL technique.

**Frequency-upconverted photoluminescence (PL) and time-resolved PL excited by simultaneous multi-photon absorption, determination of multi-photon absorption cross-sections, and spectral overlap between shell emission and excitation of core and 2PA property of the $(OA)_2PbBr_4$ nanoplates (NPs)**

Multi-photon excited upconversion PL measurements have been demonstrated to provide much higher detection sensitivity for characterizing MPA properties of both inorganic semiconductor NCs and organic molecules than open-aperture *Z*-scan, especially for higher-order MPA and highly luminescent MPA-active materials[17,32,33]. Due to the low detection efficiency, extremely high excitation peak intensities are required in open-aperture *Z*-scan for characterizing the high-order multi-photon absorption (*i.e.,* four- and five-photon absorption), which may cause sample damage or introduce other effects (or artifacts) with the extremely high excitation intensities. Consequently, most of the characterizations for the high-order multiphoton absorption (*i.e.,* four- and five-photon absorption) were performed with multi-photon excited upconversion PL measurements[33-35] and open-aperture *Z*-scan has been seldom used in this region. Hence, the multi-photon excited upconversion PL measurements have applied to characterize the multi-photon (2-, 3-, 4-, 5-photon) absorption cross-sections of the perovskite NCs.

Multi-photon excited photoluminescence (MEPL) from $MAPbBr_3$, $MAPbBr_3/(OA)_2PbBr_4$ and $CsPbBr_3$ NCs dispersed in toluene were investigated at room temperature by utilizing femtosecond laser pulses (~ 50 fs, 1 kHz) with wavelengths ranging from 675 to 2300 nm for excitation. The dilute toluene solution of the NCs contained in 2-mm-thick quartz cuvettes was employed for the MEPL measurements. The excitation laser pulses (~50 fs, 1 kHz, 250-2600 nm) were generated by an optical parametric amplifier (OPA) (OperA-Solo, Coherent) pumped by the regenerative amplified femtosecond Ti:Sapphire laser system (~ 50

fs, 800 nm, 1 kHz,; Libra, Coherent). For excitation wavelengths smaller than 800 nm, a notch filter at 800 nm was used to cut-off light at undesired wavelengths. For excitation wavelengths larger than 800 nm, the OPA output was further filtered by a suitable filter (long-pass filter at 750 nm or 1500 nm) to remove unwanted stray light. Supplementary Figure 5 displays a schematic diagram of the experimental set-up. A circular lens with focal length of 20 cm was applied to focus the laser pulses onto the perovskite nanocrystals in toluene solution. The NC samples were placed 23.5 cm away from the lens (3.5 cm away from the focal point) to avoid the high excitation peak intensity on the samples and to have larger excitation area (thus larger frequency-upconverted signal). Two continuously variable neutral density filters were employed to adjust the incident energy of laser pulses. Frequency-upconverted PL from the nanocrystals was collected at a backscattering angle of $150^o$ utilizing a pair of lenses into an optical fibre which was coupled into a spectrometer (Acton, Spectra Pro 2750i) and detected by a charge coupled device (Princeton Instruments, Pixis 100B). A short-pass filter (650 nm) was employed to filter out the scattered light at the excitation laser frequencies

The 1PA spectra of the NCs in Supplementary Figs 1a-c reveal absorption maxima at ~ 505 nm & ~ 510 nm, and negligible absorbance in wavelengths longer than ~ 530 nm. Consequently, the PL spectra from the NCs acquired at 800, 1200, 1600, and 2100 nm excitation illustrated in Supplementary Figs 6a-c and Fig. 1b in main text correspond to the two-, three-, four- and five-photon absorption processes (excitation fluence ranges ~ 0.33 – 1.16 mJ $cm^{-2}$, ~ 0.66 – 1.82 mJ $cm^{-2}$, ~ 1.16 – 2.31 mJ $cm^{-2}$, ~ 3.30 – 5.61 mJ $cm^{-2}$; larger upconversion PL intensity in lower-order multi-photon processes even at lower excitation fluence). Moreover, the nearly quadratic, cubic, biquadratic and quintic excitation fluence dependence of the spectrally integrated PL intensity shown in the insets of Supplementary Figs 6a-cand Fig. 1b with excitation fluence ranges ~ 0.33 – 1.16 mJ $cm^{-2}$, ~ 0.66 – 1.82 mJ $cm^{-2}$, ~ 1.16 – 2.31 mJ $cm^{-2}$, ~ 3.30 – 5.61 mJ $cm^{-2}$ further justify that MPA is responsible for the fluorescence excitation at infrared wavelengths in these NCs. Supplementary Figure 6d and Figure 1c in main text schematically illustrate the corresponding

frequency-upconverted PL processes from simultaneous 2-, 3-, 4- and 5PA excitation via virtual energy levels. Photographs of frequency-upconverted PL from the NCs collected at 800, 1200, 1600, 2100 nm displayed in Supplementary Fig. 11 provide more direct evidence for the occurrence of MEPL processes. As in Supplementary Fig. 11, the core-shell $MAPbBr_3/(OA)_2PbBr_4$ NCs (~2.1 μM in toluene) apparently exhibit much brighter frequency-upconverted PL than the core-only $MAPbBr_3$ NCs (~2.0 μM in toluene). Despite the $CsPbBr_3$ NCs have a lower PLQY and smaller concentration, the frequency-upconverted PL from the $MAPbBr_3$ and $CsPbBr_3$ NCs (~1.0 μM in toluene) exhibit similar brightness. This is because the more strongly confined $CsPbBr_3$ NCs possess larger MPA cross-sections than $MAPbBr_3$ NCs.

To exemplify the superior five-photon excited upconversion PL of the perovskite NCs, we have also performed a direct comparison between the five-photon excited PL from $MAPbBr_3/(OA)_2PbBr_4$ NCs (~2.1 μM) and from R6G (~ 2.1 μM) under the same experimental conditions. R6G has been well demonstrated to be a nonlinear optical active organic dye with relatively large multi-photon absorption cross-sections and high PLQY[36,37]. Since there have been no report on the five-photon absorption in R6G, we only provide a qualitatively direct comparison on the five-photon excited PL photos. The unedited photos (taken with the same camera and the same exposure) displayed in Supplementary Fig. 12 show the five-photon excited PL from $MAPbBr_3/(OA)_2PbBr_4$ NCs in toluene as compared to R6G in methanol at 2100 nm femtosecond laser excitation. As in Supplementary Fig. 12, under the applied excitation conditions, no five-photon excited PL was observed from the R6G in methonal and no upconversion PL signal could be detected with our visible monochromator (Acton, Spectra Pro 2750i) coupled with CCD. In contrast, relatively bright five-photon excited PL was excited under the same experimental conditions at 2100 nm, validating the much superior five-photon absorption properties of our $MAPbBr_3/(OA)_2PbBr_4$ NCs than R6G. Moreover, R6G exhibits one-photon absorption peak at around 525nm and 2100 nm is the boundary wavelength for R6G where an admixture of contributions from both the 4PA and 5PA processes is possible. This further confirms the outstanding 5PA properties of the $MAPbBr_3/(OA)_2PbBr_4$ NCs.

Comparison between the MEPL from $MAPbBr_3$, $MAPbBr_3/(OA)_2PbBr_4$ and $CsPbBr_3$ NCs under the same excitation fluence at 800, 1200, 1600 and 2100 nm displayed in Supplementary Fig. 7 clearly indicates the much larger MEPL in the core-shell $MAPbBr_3/(OA)_2PbBr_4$ NCs than the core-only $MAPbBr_3$ NCs, consistent with photos shown in Supplementary Fig. 11. $CsPbBr_3$ NCs with lower PLQY and smaller concentration display MEPL with similar amplitude to $MAPbBr_3$ NCs as presented in Supplementary Fig. 7 (under the same irradiation fluence), in accordance with photos in Supplementary Fig. 11 and demonstrating its larger MPA cross-sections. The normalized one-photon excited PL spectra and MEPL spectra of the NCs are displayed in Supplementary Fig. 10, for comparison. As illustrated, the MEPL spectra are on the red side of the one-photon excited PL. Such red-shift of the MEPL spectra with respect to one-photon counterpart has been well reported in traditional semiconductor NCs[38] and can be ascribed to the reabsorption effect and size inhomogeneity[39,40].

We applied the MEPL measurements on the $MAPbBr_3$, $MAPbBr_3/(OA)_2PbBr_4$ and $CsPbBr_3$ NCs at wavelengths ranging from 675 to 2300nm using the measured $\sigma_2$ values at 800 nm as a standard to determine their MPA cross-sections ($\sigma_n$), thus acquiring the MPA spectra. Through integrating $\Delta f_n$ given by Supplementary equation 4 over the entire laser focused volume and time, we quantify and obtain the MEPL strength $F_n$[15,16].

$$F_n = \iiint \Delta f_n = \iiint (1/n) \emptyset \rho \eta \sigma_n [I_i(r,z,t)]^n / (\hbar\omega)^n dsdzdt \qquad \text{(Supplementary equation 4)}$$

In Supplementary equation 4, factor $1/n$ denotes the fact that $n$-photons are simultaneously absorbed from the infrared incident laser beam for generating each fluorophore excitation. $\emptyset$ is MEPL collection efficiency of the experimental setup, $\rho$ is molar concentration of NCs, $\eta$ is PLQY and the product of $\eta$ and $\sigma_n$ is multi-photon action cross-section, a direct measurement of MEPL brightness. $I_i(r,z,t)$ is the incident laser intensity at the small volume of $ds \cdot dz$ in sample at time $t$. The beam waist at the sample point is $w(d) = w_0\sqrt{1 + d^2/z_0^2}$. $d$ is the distance of the sample to the focal point. $w_0$ is the beam waist of laser beam at the

focal point. $z_0$ is the Rayleigh length and is related to $w_0$ by $z_0 = \pi w_0^2/\lambda_0$. $w_0$ is related to the incident laser beam waist before focal lens $w_0^{'}$ by[41] $w_0 \approx \lambda_0 f/(n\pi w_0^{'})$, which is related to the laser wavelength $\lambda_0$. $f$ is the focal length, $n$ is the refractive index of the air $n \approx 1$. Taking account of the fact that NCs contained in 2-mm-thick quartz cuvettes were placed 3.5 cm away from focal point of the lens (beam waists at the sample point are dependent on the excitation wavelengths) and considering the spatial and temporal profiles of the laser pulses are Gaussian functions, the MEPL signal $F_n$ (n = 2, 3, 4, and 5) for 2PA, 3PA, 4PA and 5PA processes can be derived as:

$$F_2 \approx \pi^{3/2}\emptyset\rho\eta\sigma_2 L\tau_p\omega_0^2 d^2 I_0^2/[8\sqrt{2}z_0^2(\hbar\omega)^2] \quad \text{(Supplementary equation 5)}$$

$$F_3 \approx \pi^{3/2}\emptyset\rho\eta\sigma_3 L\tau_p\omega_0^2 d^2 I_0^3/[18\sqrt{3}z_0^2(\hbar\omega)^3] \quad \text{(Supplementary equation 6)}$$

$$F_4 \approx \pi^{3/2}\emptyset\rho\eta\sigma_4 L\tau_p\omega_0^2 d^2 I_0^4/[64z_0^2(\hbar\omega)^4] \quad \text{(Supplementary equation 7)}$$

$$F_5 \approx \pi^{3/2}\emptyset\rho\eta\sigma_5 L\tau_p\omega_0^2 d^2 I_0^5/[50\sqrt{5}z_0^2(\hbar\omega)^5] \quad \text{(Supplementary equation 8)}$$

, respectively. In which the approximation of $1 + d^2/z_0^2 \approx d^2/z_0^2$ is taken with the fact that $d^2/z_0^2 \gg 1$. $d$ is distance between sample and focal point of the lens (3.5 cm). $\tau_p$ is laser pulse width at $e^{-1}$ maximum, $I_0$ is laser intensity at the sample position.

Through applying the above expressions of $F_n$ ($n$ = 2, 3, 4, and 5) (Supplementary equations 4-7) and utilizing the $\sigma_2$ values at 800 nm measured by $Z$-scan as the standard, the $\sigma_n$ ($n$ = 2, 3, 4, and 5) values of $MAPbBr_3$, $MAPbBr_3/(OA)_2PbBr_4$ and $CsPbBr_3$ NCs can be obtained over the wavelength range spanning from 675 – 2300 nm, as shown in Fig. 1d and Figs 2a-c in the main text.

A similar experimental set-up to that of multi-photon excited photoluminescence measurements was utilized for the time-resolved PL measurements. Instead of using the CCD for PL detection, the one-photon-excited and multi-photon-excited PL emission were acquired by a Optronis Optoscope streak camera system, which possesses an ultimate temporal resolution of ~10 ps. Importantly, comparison between the one-photon excited PL decay traces in $MAPbBr_3$ and $MAPbBr_3/(OA)_2PbBr_4$ NCs displayed in

Supplementary Fig. 13a clearly demonstrates the prolonged PL lifetime in the core-shell NCs. Moreover, a prolonged five-photon excited PL lifetime of the $MAPbBr_3/(OA)_2PbBr_4$ NCs compared to the core-only counterpart was observed and depicted in Supplementary Fig. 13b. The increased PL lifetime from one- and multi-photon excitation as well as the enhanced PLQY and stability[3] in $MAPbBr_3/(OA)_2PbBr_4$ NCs stem from the effective surface passivation provided by the $(OA)_2PbBr_4$ shell. This results in the reduction of the nonradiative surface traps, thus increasing the multi-photon transition probability[42,43]. Besides, the longer one- and multi-photon excited PL lifetime in $MAPbBr_3/(OA)_2PbBr_4$ NCs, a good spectral overlap between the shell emission and the excitation of the core (Supplementary Fig. 13c) and the 2PA of the $(OA)_2PbBr_4$ NPs as shown in Supplementary Fig. 14 suggests that the nonradiative energy transfer from the shell to the core through antenna-like effect could also contribute to the observed enhanced $\sigma_n$ in the core-shell $MAPbBr_3/(OA)_2PbBr_4$ NCs[44,45]. The effective two-photon absorption coefficient of the $(OA)_2PbBr_4$ NPs solution in toluene is determined to be ~ 0.17 cm $GW^{-1}$ through utilizing Supplementary equation 2. The two-photon absorption cross-section of the $(OA)_2PbBr_4$ NPs cannot be obtained due to the unknown concentration of $(OA)_2PbBr_4$ NPs in toluene, and it is beyond the scope of this work.

## Supplementary References


1. Wang, Y. *et al.* Nonlinear absorption and low-threshold multiphoton pumped stimulated emission from all-inorganic perovskite nanocrystals. *Nano Lett.* **16**, 448-453 (2016).
2. Xu, Y. *et al.* Two-photon-pumped perovskite semiconductor nanocrystal lasers. *J. Am. Chem. Soc.* **138**, 3761-3768 (2016).
3. Bhaumik, S. *et al.* Highly stable, luminescent core-shell type methylammonium-octylammonium lead bromide layered perovskite nanoparticles. *Chem. Commun.* **52**, 7118-7121 (2016).
4. Tyagi, P., Arveson, S. M. & Tisdale, W. A. Colloidal organohalide perovskite nanoplatelets exhibiting quantum confinement. *J. Phys. Chem. Lett.* **6**, 1911-1916 (2015).
5. Zhang, M. *et al.* Composition-dependent photoluminescence intensity and prolonged recombination lifetime of perovskite $CH_3NH_3PbBr_{3-x}Cl_x$ films. *Chem. Commun.* **50**, 11727-11730 (2014).
6. Stoumpos, C. C. *et al.* Crystal growth of the perovskite semiconductor $CsPbBr_3$: a new material for high-energy radiation detection. *Cryst. Growth Des.* **13**, 2722-2727 (2013).
7. Tanaka, K. *et al.* Comparative study on the excitons in lead-halide-based perovskite-type crystals $CH_3NH_3PbBr_3$ $CH_3NH_3PbI_3$. *Solid State Commun.* **127**, 619-623 (2003).
8. Mitzi, D. B. Templating and structural engineering in organic-inorganic perovskites. *J. Chem. Soc., Dalton Trans.* 1-12 (2001).
9. Blöchl, P. E. Projector augmented-wave method. *Phys. Rev. B* **50**, 17953-17979 (1994).
10. Perdew, J. P., Burke, K. & Ernzerhof, M. Generalized gradient approximation made simple. *Phys. Rev. Lett.* **77**, 3865-3868 (1996).
11. Kresse, G. & Furthmüller, J. Efficient iterative schemes for \textit{ab initio} total-energy calculations using a plane-wave basis set. *Phys. Rev. B* **54**, 11169-11186 (1996).
12. Monkhorst, H. J. & Pack, J. D. Special points for Brillouin-zone integrations. *Phys. Rev. B* **13**, 5188-5192 (1976).
13. Xu, Q., Fan, W. & Kuo, J.-L. The natural valence band offset of dilute $GaAs_{1-x}N_x$ and GaAs: the first-principles approach. *Comput. Mater. Sci.* **49**, S150-S152 (2010).
14. Rulliere. C. *Femtosecond Laser Pulses: Principles and Experiments*. Springer (1998).
15. Xu, C. & Webb, W. W. Measurement of two-photon excitation cross sections of molecular fluorophores with data from 690 to 1050 nm. *J. Opt. Soc. Am. B* **13**, 481-491 (1996).
16. Maiti, S., Shear, J. B., Williams, R. M., Zipfel, W. R. & Webb, W. W. Measuring serotonin distribution in live cells with three-photon excitation. *Science* **275**, 530-532 (1997).
17. Arnaud, J. A. *et al.* Technique for fast measurement of Gaussian laser beam parameters. *Appl. Opt.* **10**, 2775-2776 (1971).
18. Khosrofian, J. M. & Garetz, B. A. Measurement of a Gaussian laser beam diameter through the direct inversion of knife-edge data. *Appl. Opt.* **22**, 3406-3410 (1983).
19. de Araújo, M. A., Silva, R., de Lima, E., Pereira, D. P. & de Oliveira, P. C. Measurement of Gaussian laser beam radius using the knife-edge technique: improvement on data analysis. *Appl. Opt.* **48**, 393-396 (2009).
20. Sheik-Bahae, M., Said, A. A., Wei, T. H., Hagan, D. J. & Van Stryland, E. W. Sensitive measurement of optical nonlinearities using a single beam. *Ieee J. Quantum Elect.* **26**, 760-769 (1990).
21. Sheik-Bahae, M., Hutchings, D. C., Hagan, D. J. & Van Stryland, E. W. Dispersion of bound electron nonlinear refraction in solids. *Ieee J. Quantum Elect.* **27**, 1296-1309 (1991).
22. Krauss, T. D. & Wise, F. W. Femtosecond measurement of nonlinear absorption and refraction in CdS, ZnSe, and ZnS. *Appl. Phys. Lett.* **65**, 1739-1741 (1994).
23. Walters, G. *et al.* Two-photon absorption in organometallic bromide perovskites. *ACS Nano* **9**, 9340-9346 (2015).
24. Blanton, S. A., Dehestani, A., Lin, P. C. & Guyot-Sionnest, P. Photoluminescence of single

semiconductor nanocrystallites by two-photon excitation microscopy. *Chem. Phys. Lett.* **229**, 317-322 (1994).

25. Saidaminov, M. I. *et al.* High-quality bulk hybrid perovskite single crystals within minutes by inverse temperature crystallization. *Nat. Commun.* **6**, 7586 (2015).
26. Nikesh, V. V. *et al.* Optical nonlinearity of monodispersed, capped ZnS quantum particles. *Appl. Phys. Lett.* **84**, 4602-4604 (2004).
27. Lad, A. D., Prem Kiran, P., Ravindra Kumar, G. & Mahamuni, S. Three-photon absorption in ZnSe and ZnSe⁄ZnS quantum dots. *Appl. Phys. Lett.* **90**, 133113 (2007).
28. Woodall, M. A. *Nonlinear Absorption Techniques and Measurements in Semiconductors*. North Texas State University (1985).
29. Brandi, H. S.& de Araujo, C. B. Multiphoton absorption coefficients in solids: a universal curve. *J. Phys. C: Solid State Phys.* **16**, 5929-5936 (1983).
30. He, J., Qu, Y., Li, H., Mi, J. & Ji, W. Three-photon absorption in ZnO and ZnS crystals. *Opt. Express* **13**, 9235-9247 (2005).
31. Sutherland, R. L. *Handbook of Nonlinear Optics 2nd edn*. CRC Press (2003).32. Xu, C., Zipfel, W., Shear, J. B., Williams, R. M. & Webb, W. W. Multiphoton fluorescence excitation: new spectral windows for biological nonlinear microscopy. *Proc. Natl. Acad. Sci. U.S.A.* **93**, 10763-10768 (1996).
33. He, G. S., Tan, L.-S., Zheng, Q. & Prasad, P. N. Multiphoton absorbing materials: molecular designs, characterizations, and applications. *Chem. Rev.* **108**, 1245-1330 (2008).
34. He, G. S. *et al.* Two-, three-, and four-photon-pumped stimulated cavityless lasing properties of ten stilbazolium-dyes solutions. *J. Opt. Soc. Am. B* **22**, 2219-2228 (2005).
35. Fan, H. H., Guo, L., Li, K. F., Wong, M. S. & Cheah, K. W. Exceptionally strong multiphoton-excited blue photoluminescence and lasing from ladder-type oligo(p-phenylene)s. *J. Am. Chem. Soc.* **134**, 7297-7300 (2012).
36. Albota, M. A., Xu, C. & Webb, W. W. Two-photon fluorescence excitation cross sections of biomolecular probes from 690 to 960 nm. *Appl. Opt.* **37**, 7352-7356 (1998).
37. Makarov, N. S., Drobizhev, M. & Rebane, A. Two-photon absorption standards in the 550-1600 nmexcitation wavelength range. *Opt. Express* **16**, 4029-4047 (2008).
38. He, G. S. *et al.* Multi-photon excitation properties of CdSe quantum dots solutions and optical limiting behavior in infrared range. *Opt. Express* **15**, 12818-12833 (2007).
39. Wang, Y. *et al.* Stimulated emission and lasing from CdSe/CdS/ZnS core-multi-shell quantum dots by simultaneous three-photon absorption. *Adv. Mater.* **26**, 2954-2961 (2014).
40. He, T. *et al.* Wavelength dependence of optical nonlinearity of terpyridine-based Zn(II)-coordinated rigid linear polymers. *Appl. Phys. Lett.* **101**, 213302 (2012).
41. Saleh, B. E. A. & Teich, M. C. *Fundamentals of Photonics 2nd edn.* Wiley (2007).42. Zhu, B. H., Zhang, H. C., Zhang, J. Y., Cui, Y. P. & Zhou, Z. Q. Surface-related two-photon absorption and refraction of CdSe quantum dots. *Appl. Phys. Lett.* **99**, 021908 (2011).
43. Zhu, B.-H., Zhang, H.-C., Zhang, Z.-Y., Cui, Y.-P. & Zhang, J.-Y. Effect of shell thickness on two-photon absorption and refraction of colloidal CdSe/CdS core/shell nanocrystals. *Appl. Phys. Lett.* **99**, 231903 (2011).
44. Xing, G. *et al.* Enhanced tunability of the multiphoton absorption cross-section in seeded CdSe/CdS nanorod heterostructures. *Appl. Phys. Lett.* **97**, 061112 (2010).
45. He, T. *et al.* Efficient energy transfer under two-photon excitation in a 3D, supramolecular, Zn(II)-coordinated, self-assembled organic network. *Adv. Optical Mater.* **2**, 40-47 (2014).
46. Zheng, Q. *et al.* Frequency-upconverted stimulated emission by simultaneous five-photon absorption. *Nat. Photon.* **7**, 234-239 (2013).
47. He, G. S. *et al.* Two-, three-, and four-photon-pumped stimulated cavityless lasing properties of ten stilbazolium-dyes solutions. *J. Opt. Soc. Am. B* **22**, 2219-2228 (2005).

48. Hernández, F. E., Belfield, K. D., Cohanoschi, I., Balu, M. & Schafer, K. J. Three- and four-photon absorption of a multiphoton absorbing fluorescent probe. *Appl. Opt.* **43**, 5394-5398 (2004).
49. Belfield, K. D., Bondar, M. V., Przhonska, O. V. & Schafer, K. J. Steady-state spectroscopic and fluorescence lifetime measurements of new two-photon absorbing fluorene derivatives. *J. Fluoresc.* **12**, 449-454 (2002).
50. Corrêa, D. S., De Boni, L., Balogh, D. T. & Mendonça, C. R. Three- and four-photon excitation of poly(2-methoxy-5-(2′-ethylhexyloxy)-1,4-phenylenevinylene) (MEH-PPV). *Adv. Mater.* **19**, 2653-2656 (2007).
51. Quah, H. S. *et al.* Multiphoton harvesting metal-organic frameworks. *Nat. Commun.* **6**, 7954 (2015).
52. He, G. S. *et al.* Multi-photon excitation properties of CdSe quantum dots solutions and optical limiting behavior in infrared range. *Opt. Express* **15**, 12818-12833 (2007).
53. Pu, S.-C. *et al.* The empirical correlation between size and two-photon absorption cross section of CdSe and CdTe quantum dots. *Small* **2**, 1308-1313 (2006).
54. Chon, J. W. M., Gu, M., Bullen, C. & Mulvaney, P. Three-photon excited band edge and trap emission of CdS semiconductor nanocrystals. *Appl. Phys. Lett.* **84**, 4472-4474 (2004).
55. Xing, G., Ji, W., Zheng, Y. & Ying, J. Y. Two- and three-photon absorption of semiconductor quantum dots in the vicinity of half of lowest exciton energy. *Appl. Phys. Lett.* **93**, 241114 (2008).
56. He, J., Ji, W., Mi, J., Zheng, Y. & Ying, J. Y. Three-photon absorption in water-soluble ZnS nanocrystals. *Appl. Phys. Lett.* **88**, 181114 (2006).
57. Lad, A. D., Kiran, P. P., More, D., Kumar, G. R. & Mahamuni, S. Two-photon absorption in ZnSe and ZnSe⁄ZnS core/shell quantum structures. *Appl. Phys. Lett.* **92**, 043126 (2008).
58. Xing, G., Ji, W., Zheng, Y. & Y. Ying, J. High efficiency and nearly cubic power dependence of below-band-edge photoluminescence in water-soluble, copper doped ZnSe/ZnS Quantum dots. *Opt. Express* **16**, 5715-5720 (2008).
59. Xing, G., Chakrabortty, S., Ngiam, S. W., Chan, Y. & Sum, T. C. Three-photon absorption in seeded CdSe/CdS nanorod heterostructures. *J. Phys. Chem. C* **115**, 17711-17716 (2011).
60. Xing, G. *et al.* Ultralow-threshold two-photon pumped amplified spontaneous emission and lasing from seeded CdSe/CdS nanorod heterostructures. *ACS Nano* **6**, 10835-10844 (2012).
61. Zheng, Q., He, G. S., Lu, C. & Prasad, P. N. Synthesis, two- and three-photon absorption, and optical limiting properties of fluorene-containing ferrocene derivatives. *J. Mater. Chem.* **15**, 3488-3493 (2005).
62. Suo, Z., Drobizhev, M., Spangler, C. W., Christensson, N. & Rebane, A. New fluorophores based on trifluorenylamine with very large intrinsic three-photon absorption cross sections. *Org. Lett.* **7**, 4807-4810 (2005).
63. Larson, D. R. *et al.* Water-soluble quantum dots for multiphoton fluorescence imaging in vivo. *Science* **300**, 1434-1436 (2003).
64. Qu, Y. & Ji, W. Two-photon absorption of quantum dots in the regime of very strong confinement: size and wavelength dependence. *J. Opt. Soc. Am. B* **26**, 1897-1904 (2009).
65. Bol, A. A. & Meijerink, A. Luminescence quantum efficiency of nanocrystalline ZnS:$Mn^{2+}$. 1. surface passivation and $Mn^{2+}$ concentration. *J. Phys. Chem. B* **105**, 10197-10202 (2001).
66. Padilha, L. A. *et al.* Optimization of band structure and quantum-size-effect tuning for two-photon absorption enhancement in quantum dots. *Nano Lett.* **11**, 1227-1231 (2011).
67. Semonin, O. E. *et al.* Absolute photoluminescence quantum yields of IR-26 dye, PbS, and PbSe quantum dots. *J. Phys. Chem. Lett.* **1**, 2445-2450 (2010).
68. Song, J.-J., Tsen, K.-T., Betz, M. & Elezzabi, A. Y. Ultrafast phenomena in semiconductors and nanostructure materials XIV. in *Society of Photo-Optical Instrumentation Engineers (SPIE) Conference Series* **7600,** (2010).
69. Wang, Y. *et al.* Near resonant and nonresonant third-order optical nonlinearities of colloidal InP/ZnS

quantum dots. *Appl. Phys. Lett.* **102**, 021917 (2013).
70. Wawrzynczyk, D., Szeremeta, J., Samoc, M. & Nyk, M. Optical nonlinearities of colloidal InP@ZnS core-shell quantum dots probed by Z-scan and two-photon excited emission. *APL Mater.* **3**, 116108 (2015).
71. Xing, G., Jiang, J., Ying, J. Y. & Ji, W. Fe3O4-Ag nanocomposites for optical limiting: broad temporal response and low threshold. *Opt. Express* **18**, 6183-6190 (2010).
72. Albota, M. *et al.* Design of organic molecules with large two-photon absorption cross sections. *Science* **281**, 1653-1656 (1998).
73. Chung, S.-J. *et al.* Strong, low-energy two-photon absorption in extended amine-terminated cyano-substituted phenylenevinylene oligomers. *J. Am. Chem. Soc.* **127**, 10844-10845 (2005).
74. Belfield, K. D. *et al.* Linear and two-photon photophysical properties of a series of symmetrical diphenylaminofluorenes. *Chem. Mater.* **16**, 2267-2273 (2004).
75. De Boni, L. *et al.* Nonlinear absorption spectrum in MEH-PPV/chloroform solution: a competition between two-photon and saturated absorption processes. *J. Phys. Chem. B* **108**, 5221-5224 (2004).
76. Bauer, C. *et al.* Two-photon pumped lasing from a two-dimensional photonic bandgap structure with polymeric gain material. *Adv. Mater.* **14**, 673-676 (2002).
77. Harrison, M. G. *et al.* Two-photon fluorescence and femtosecond two-photon absorption studies of MeLPPP, a ladder-type poly(phenylene) with low intra-chain disorder. *Chem. Phys. Lett.* **313**, 755-762 (1999).
78. Drobizhev, M., Karotki, A., Rebane, A. & Spangler, C. W. Dendrimer molecules with record large two-photon absorption cross section. *Opt. Lett.* **26**, 1081-1083 (2001).
79. Padmawar, P. A. *et al.* Large cross-section enhancement and intramolecular energy transfer upon multiphoton absorption of hindered diphenylaminofluorene-C60 dyads and triads. *Chem. Mater.* **18**, 4065-4074 (2006).
80. Zhao, Y. *et al.* Synthesis, spectroscopic and nonlinear optical properties of multiple [60]fullerene–oligo(p-phenylene ethynylene) hybrids. *Chem. Eur. J.* **11**, 3643-3658 (2005).
81. Cho, J.-Y. *et al.* Strong two-photon absorption at telecommunications wavelengths in nickel bis(dithiolene) complexes. *Opt. Lett.* **32**, 671-673 (2007).
82. Das, S., Nag, A., Goswami, D. & Bharadwaj, P. K. Zinc(II)- and copper(I)-mediated large two-photon absorption cross sections in a bis-cinnamaldiminato Schiff base. *J. Am. Chem. Soc.* **128**, 402-403 (2006).
83. Drobizhev, M. *et al.* Extremely strong near-IR two-photon absorption in conjugated porphyrin dimers: quantitative description with three-essential-states model. *J. Phys. Chem. B* **109**, 7223-7236 (2005).
84. Ikeda, C. *et al.* Helicity induction and two-photon absorbance enhancement in zinc(II) meso−meso linked porphyrin oligomers via intermolecular hydrogen bonding interactions. *J. Am. Chem. Soc.* **127**, 534-535 (2005).
85. Kuzyk, M. G. & Dirk, C. W. *Characterization Techniques and Tabulations for Organic Nonlinear Optical Materials*. Marcel Dekker (1998).
86. Andrade, A. A. *et al.* Two-photon absorption investigation in reduced and oxidized cytochrome c solutions. *Chem. Phys. Lett.* **390**, 506-510 (2004).
